# Agronomic and Seed Quality Traits Dissected by Genome-Wide Association Mapping in *Brassica napus*

**DOI:** 10.3389/fpls.2016.00386

**Published:** 2016-03-31

**Authors:** Niklas Körber, Anja Bus, Jinquan Li, Isobel A. P. Parkin, Benjamin Wittkop, Rod J. Snowdon, Benjamin Stich

**Affiliations:** ^1^Quantitative Crop Genetics, Max Planck Institute for Plant Breeding ResearchCologne, Germany; ^2^Plant Breeding and Biotechnology, Institute of Crop Science and Resource Conservation, University of BonnBonn, Germany; ^3^Agriculture and Agri-Food CanadaSaskatoon, SK, Canada; ^4^Department of Plant Breeding, Research Centre for BioSystems, Land Use and Nutrition, Justus Liebig UniversityGiessen, Germany

**Keywords:** *Brassica napus*, agronomic traits, seed quality, genome-wide association mapping, flowering, erucic acid, marker-assisted selection, candidate genes

## Abstract

In *Brassica napus* breeding, traits related to commercial success are of highest importance for plant breeders. However, such traits can only be assessed in an advanced developmental stage. Molecular markers genetically linked to such traits have the potential to accelerate the breeding process of *B. napus* by marker-assisted selection. Therefore, the objectives of this study were to identify (i) genome regions associated with the examined agronomic and seed quality traits, (ii) the interrelationship of population structure and the detected associations, and (iii) candidate genes for the revealed associations. The diversity set used in this study consisted of 405 *B. napus* inbred lines which were genotyped using a 6K single nucleotide polymorphism (SNP) array and phenotyped for agronomic and seed quality traits in field trials. In a genome-wide association study, we detected a total of 112 associations between SNPs and the seed quality traits as well as 46 SNP-trait associations for the agronomic traits with a *P* < 1.28e-05 (Bonferroni correction of α = 0.05) for the inbreds of the spring and winter trial. For the seed quality traits, a single SNP-sulfur concentration in seeds (SUL) association explained up to 67.3% of the phenotypic variance, whereas for the agronomic traits, a single SNP-blossom color (BLC) association explained up to 30.2% of the phenotypic variance. In a basic local alignment search tool (BLAST) search within a distance of 2.5 Mbp around these SNP-trait associations, 62 hits of potential candidate genes with a BLAST-score of ≥100 and a sequence identity of ≥70% to *A. thaliana* or *B. rapa* could be found for the agronomic SNP-trait associations and 187 hits of potential candidate genes for the seed quality SNP-trait associations.

## 1. Introduction

In *Brassica napus* breeding, traits related to commercial success are of highest importance (Friedt and Snowdon, 2010). However, such traits in many cases can only be assessed in an advanced developmental stage. Therefore, the use of marker-assisted selection (MAS) has the potential to save time in the breeding process and increase the gain of selection. In order to do so, the identification of quantitative trait loci (QTL) controlling these traits is required. However, the detection of QTL which explain an adequate percentage of the phenotypic variance is challenging.

Linkage mapping or association mapping approaches are suitable methods for the discovery of QTL. Various studies in *B. napus* have identified several QTL for agronomic and seed quality traits using such approaches. Würschum et al. (2012) detected in nine segregating populations of elite rapeseed inbreds several QTL for diverse traits, including flowering time, plant height, protein content, oil content, glucosinolate content, and grain yield. Udall et al. (2006) used two DH populations and detected genomic regions which contributed to variation of grain yield, days to flowering, and leaf blight disease resistance. Due to only two parental alleles and large confidence intervals of QTL, however, the results of linkage mapping studies had so far little impact on the breeding process (Van Inghelandt et al., 2012).

Hasan et al. (2008) identified in an association mapping study with *B. napus* germplasm simple sequence repeat (SSR) markers which were physically linked to candidate genes for glucosinolate biosynthesis in *Arabidopsis thaliana*, to be associated with variation of the seed glucosinolate content in *B. napus*. However, the results of Linkage disequilibrium (LD) analyses suggested that the number of such SSR-markers is at the lower end of what is required to have a high power to detect marker-phenotype associations for seed quality traits in rapeseed (Bus et al., 2011; Delourme et al., 2013). In the meantime the *B. napus* A genome sequence from *B. rapa* (Wang et al., 2011b) as well as the C genome sequence from *B. oleracea* were published (Yu et al., 2013). This information allowed the design and use of a 6K SNP chip and latterly a 60K SNP chip. Bus et al. (2014) identified 29 loci significantly associated with variation of the shoot ionome in our diversity set consisting of 509 inbred lines that was genotyped with the 6K SNP array. Furthermore, in a previous study 63 significant associations for seedling development traits and 31 SNP-gene associations for candidate genes related to seedling development were identified using the same 6K SNP array (Körber et al., 2015). Recently, Li et al. (2014), Luo et al. (2015), and Hatzig et al. (2015) used the 60K SNP array and identified in an association mapping study in different *B. napus* populations significant associations for seed weight and seed quality traits, harvest index as well as seed germination and vigor traits.

In this study, we performed a genome-wide association study (GWAS) in our large-size worldwide diversity set of 405 *B. napus* inbred lines and analyzed 15 agronomic as well as 15 seed quality traits with a sufficient number of SNP markers which were mapped to the *B. napus* sequence.

The objectives of our study were to identify (i) genome regions associated with the examined agronomic and seed quality traits, (ii) the interrelationship of population structure and the detected associations, and (iii) candidate genes for the revealed associations.

## 2. Materials and methods

### 2.1. Plant material and field experiments

A subset of 405 rapeseed inbreds from the diversity set examined by Bus et al. (2011) was used in this study. The accessions belong to eight different germplasm types, namely winter oilseed rape (OSR) (156), winter fodder (8), swede (51), semi-winter OSR (7), spring OSR (177), spring fodder (4), and vegetable (2).

The multiplication of the genotypes was done in such a way that maternal environmental effects were minimized. The *B. napus* diversity set was evaluated in field experiments for several agronomic traits, and the harvested seeds were analyzed by near infrared reflectance spectroscopy (NIRS) to extract the seed quality parameters MOI, OIL, PRT, GSL, SUL, OLA, LIA, and ERA according to the standard protocol of VDLUFA and the parameters NDF, ADF, and ADL according to Wittkop et al. (2012) (Table 1).

**Table 1:** **Traits assessed in the ***B. napus*** diversity set, where h^**2**^ is the repeatability, ***R***^**2**^ the proportion of the phenotypic variance explained by population structure, and Obs. the number of replicates or location-replicate combinations in which the corresponding trait was recorded**.

**Traits**	**Abbreviation**	**Unit of measurement**	**Winter trial**			**Spring trial**	
			**Obs**.	**h^2^**	***R*^2^ (WR-MCLUST)**	**Obs**.	**h^2^**
**AGRONOMIC TRAITS**							
Emergence	EMR	1 = bad, 9 = very good	4	0.74	0.71	2	0.57
Development after emergence	DAE	1 = bad, 9 = very good	6	0.82	0.74	4	0.56
Stem elongation before winter	SAW	1 = no, 9 = much	3	0.62	0.53		
Winter hardiness	WIH	1 = bad, 9 = very good	6	0.74	0.58		
Phoma at leaves	PHO	1 = healthy, 9 = infected	2	0.41	0.02		
Lodging before flowering	LOF	1 = low, 9 = very high	3	0.41	0.50	2	0.85
Beginning of flowering	BOF	1 = early, 9 = late	7	0.94	0.79	4	0.81
Blossom color	BLC	1 = white, 3 = dark yellow	7	0.60	0.70		
End of flowering	EOF	1 = early, 9 = late	4	0.84	0.51	3	0.62
Maturity date	MYD	1 = bad, 9 = very good	2	0.26	0.17		
Lodging at maturity	LOM	1 = low, 9 = very high	5	0.58	0.30	2	0.76
Plant height	PTH	cm	6	0.68	0.15	6	0.81
Disease status before harvest	DBH	1 = healthy, 9 = infected	3	0.56	0.32	2	0.67
Phoma at harvest	PHM	1 = healthy, 9 = infected	2	0.28	0.17		
Yield	DTH	dt/ha	2	0.70	0.68	2	0.86
**SEED QUALITY TRAITS**							
Thousand grain weight	TGW	g	6	0.87	0.65		
Average projected seed area	AVA	cm^2^	3	0.81	0.59		
Moisture content	MOI	% of dry mass	4	0.61	0.34	6	0.45
Oil content	OIL	% of dry mass	7	0.86	0.53	6	0.79
Protein content	PRT	% of dry mass	5	0.81	0.46	6	0.69
Glucosinolate concentration	GSL	micromoles/g	7	0.96	0.22	6	0.97
Sulfur concentration	SUL	% of dry mass	3	0.96	0.23	6	0.96
Oleic acid concentration	OLA	% of total fatty acid	4	0.92	0.10	6	0.90
Linolenic acid concentration	LIA	% of total fatty acid	4	0.31	0.03	6	0.70
Erucic acid concentration	ERA	% of total fatty acid	6	0.96	0.09	6	0.96
Neutral detergent fiber concentration	NDF	% of dry mass	3	0.91	0.38	6	0.95
Acid detergent fiber concentration	ADF	% of dry mass	4	0.85	0.10	6	0.91
Hemicellulose concentration	HCL	% of dry mass	3	0.79	0.00	6	0.60
Acid detergent lignin concentration	ADL	% of dry mass	4	0.82	0.16	6	0.95
Cellulose concentration	CEL	% of dry mass	3	0.64	0.17	6	0.33

*For details see Materials and Methods*.

As described by Körber et al. (2012), a subset of 217 winter *B. napus* genotypes was grown in a field experiment in the growing season 2009–2010, which is designated in the following as winter trial. In 2010, a subset of 188 spring *B. napus* genotypes was evaluated at three locations in Germany with two replications per location. The experiment is designated in the following as spring trial. The phenotypic mean values of agronomic and seed quality traits of the winter and spring trials are listed in the Data Sheet S1.

### 2.2. Genotyping of SNP markers

For the GWAS, 398 *B. napus* inbred lines were assayed at Agriculture and Agri-Food Canada using a customized *B. napus* 6K Illumina Infinium SNP array (http://aafc-aac.usask.ca/ASSYST/). As described in detail in Körber et al. (2015), this array was designed from next generation sequence (NGS) data. It contained 5506 successful bead types representing the same number of potential SNPs. Samples were prepared and assayed as per the Infinium HD Assay Ultra Protocol (Infinium HD Ultra User Guide 11328087_RevB, Illumina, Inc., San Diego, CA). The Brassica 6K BeadChips were imaged using an Illumina HiScan system, and the SNP alleles were called using the Genotyping Module v1.9.4, within the GenomeStudio software suite v2011.1 (Illumina, Inc. San Diego, CA). Only SNPs with a percentage of missing data <30% across all genotypes and a minor allele frequency>0.05 as well as genotypes with a percentage of missing data < 20% across all SNPs were used for the following statistical analysis. From these 3910 SNPs, 3466 could be assigned to a physical map position derived from the reference information of the *B. napus* winter line Darmor-bzh (Chalhoub et al., 2014) (Data Sheet S2).

### 2.3. Statistical analyses

#### 2.3.1. Genome positions of trait related candidate genes

A basic local alignment search tool (BLAST) search (Altschul et al., 1990) was performed with BLASTN (*E*-value ≤ 1e-03) between the reference sequences of potential *A. thaliana* as well as *B. rapa* genes and the reference sequences of *B. napus* (v4.1) (Chalhoub et al., 2014). All positions were used which had a Bit-score ≥ 100 and a BLAST identity ≥ 70%. The gene reference sequences are either based on the five *A. thaliana* chromosome sequences NC_003070.9, NC_003071.7, NC_003074.8, NC_003075.7, NC_003076.8, or on the *B. rapa* reference sequence GCF_000309985.1.

#### 2.3.2. Adjusted entry means and principal component analysis

The adjusted entry means (**AEM**) of each genotype-trait combination, which were the basis for all further analyses, were calculated for the agronomic and seed quality traits from the winter trial using model (1) and the spring trial using model (2):

(1) $$\begin{array}{r} y_{ijm}=\mu +g_{i}+l_{j}+b_{jm}+e_{ijm} \end{array}$$

(2) $$y_{ijkm}=\mu +g_{i}+l_{j}+g_{i}*l_{j}+r_{jk}+b_{jkm}+e_{ijkm},$$

where **y**_*ijm*_ was the observation of the **i**th genotype in the **m**th block at the **j**th location, μ an intercept term, **g**_*i*_ the genotypic effect of the **i**th genotype, **l**_*j*_ the effect of the **j**th location, **b**_*jm*_ the effect of the **m**th block at the **j**th location, **e**_*ijm*_ the residual, **y**_*ijkm*_ the observation of the **i**th genotype in the **m**th block of the **k**th replication at the **j**th location, **g**_i_^*^l_*j*_ the interaction effect of the **i**th genotype and the **j**th location, **r**_*jk*_ the effect of the **k**th replicate at the **j**th location, **b**_*jkm*_ the effect of the **m**th block in the **k**th replicate of the **j**th location, and **e**_*ijkm*_ the residual.

The repeatability *h*^2^ was calculated for the various traits according to Emrich et al. (2008). Using a principal component analysis (PCA) based on 89 SSR marker data for 398 of the 405 inbreds described by Bus et al. (2011) the 214 rapeseed inbreds of the winter trial were assigned to two clusters (WR-MCLUST groups 1 and 2), whereas no distinct clusters were observed for the 184 inbreds from the spring trial.

#### 2.3.3. Assessment of linkage disequilibrium

In order to determine the physical map distance in which LD decays in our *B. napus* diversity set, *r*^2^ (the square of the correlation of the allele frequencies between all pairs of linked SNP loci) was calculated, where linked loci were defined as loci located on the same chromosome, and plotted against the physical distance in megabase pairs. The overall decay of LD was evaluated by nonlinear regression of *r*^2^ according to Hill and Weir (1988). The percentage of linked loci in significant LD was determined with the significance threshold of the 95% quantile of the *r*^2^-value among unlinked loci pairs, where unlinked loci were defined as loci located on different chromosomes. Pairwise modified Roger's distance (MRD) estimates between all inbreds and the WR-MCLUST groups 1–2 were calculated according to Wright (1978).

#### 2.3.4. GWAS - multiple forward regression

The genome-wide association analyses of the agronomic and seed quality traits were performed as a multiple forward regression analysis (Van Inghelandt et al., 2012) to take into account the LD between SNPs to identify those SNP marker combinations which explain best the genotypic variation. The Bonferroni correction (α = 0.05) was used as a P-to-enter criterion. We added the SNP with the lowest *P*-value in the single marker analysis, as fixed cofactor in the analyses, when examining all remaining SNP markers for their association with the phenotype. For each of the 30 traits, this procedure was repeated until no more significant SNPs could be selected. The above mentioned single marker analysis was based on the *PK* method (Stich et al., 2008):

(3) $$\begin{array}{l} M_{lm}=\mu +a_{m}+\overset{z}{\underset{u=1}{\sum }}P_{lu}v_{u}+g_{l}^{*}+e_{lm}, \end{array}$$

where *M*_*lm*_ was the adjusted entry mean of the **l**th inbred carrying allele *m*, a_*m*_ the effect of the **m**th allele, *v*_*u*_ the effect of the **u**th column of z columns of the population structure matrix *P, g*${}_{l}^{*}$ the residual genetic effect of the **l**th entry, and *e*_*lm*_ the residual. The first and second principal component calculated based on the 89 SSR markers (Bus et al., 2011) was used as *P* matrix. The variance of the random effect g^*^ = {$g_{1}^{*}$, …, $g_{l}^{*}$} was assumed to be Var(g^*^) = 2*K*σ${}_{g^{*}}^{2}$, where σ${}_{g^{*}}^{2}$ was the residual genetic variance. The kinship coefficient *K*_*ij*_ between inbreds *i* and *j* were calculated based on the above mentioned SSR markers according to:

(4) $$\begin{array}{l} K_{ij}=\frac{S_{ij\;-\;1}}{1+T}+1, \end{array}$$

where *S*_*ij*_ was the proportion of marker loci with shared variants between inbreds *i* and *j* and *T* the average probability that a variant from one parent of inbred *i* and *a* variant from one parent of inbred *j* are alike in state, given that they are not identical by descent (Bernardo, 1993). The optimum *T*-value was calculated according to Stich et al. (2008) for each trait. To perform the above outlined association analysis, the R package EMMA (Kang et al., 2008) was used. We chose as a significance threshold the Bonferroni correction (α = 0.05). The association analysis was performed for the inbreds of the spring trial, the inbreds of the winter trial, and for each of the two WR-MCLUST groups. For the separate association analyses of the two WR-MCLUST groups, only the kinship matrix *K* but no *P* matrix was considered.

If not stated differently, all analyses were performed with the statistical software R (R Development Core Team, 2011).

## 3. Results

**Figure 1:**
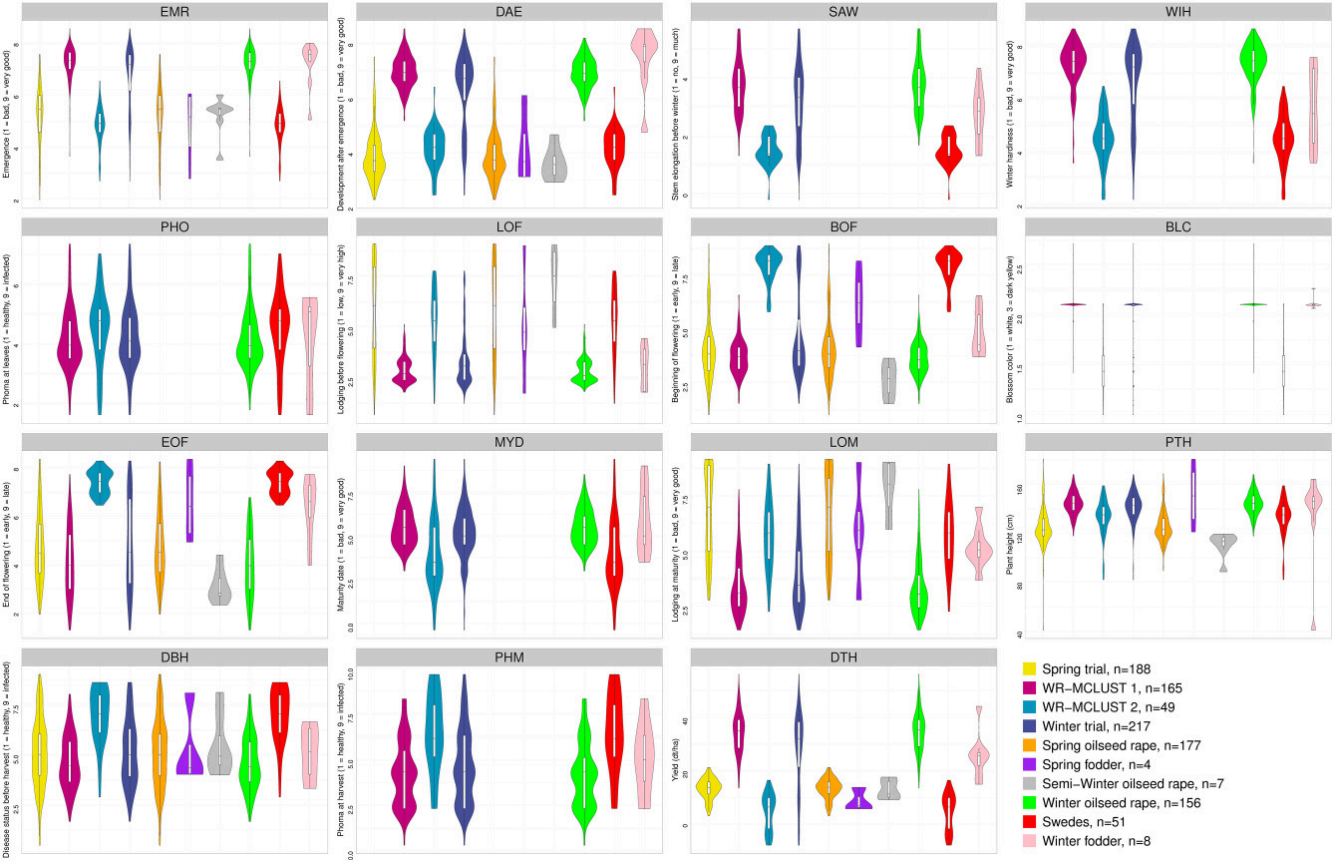
**Frequency distribution of adjusted entry means determined for 15 agronomic traits of the ***B. napus*** inbred lines for the spring and winter trial as well as the two WR-MCLUST groups and for six different germplasm types represented by different colors**. Yellow plots represent the 188 inbreds of the spring trial and blue plots the 217 inbreds of the winter trial. The number of genotypes for each germplasm type is given in the legend. In each plot, a marker denotes the median of the data, a box indicates the interquartile range, and spikes extend to the upper and lower adjacent values, overlaid is the density.

**Figure 2:**
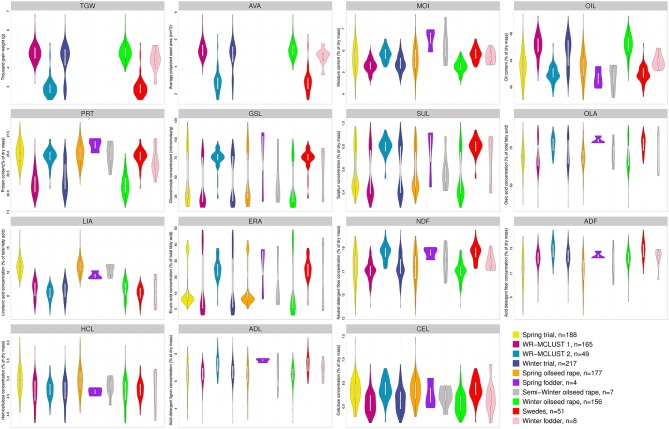
**Frequency distribution of adjusted entry means determined for 15 seed quality traits of the ***B. napus*** inbred lines for the spring and winter trial as well as the two WR-MCLUST groups and for six different germplasm types represented by different colors**. Yellow plots represent the 188 inbreds of the spring trial and blue plots the 217 inbreds of the winter trial. The number of genotypes for each germplasm type is given in the legend. In each plot, a marker denotes the median of the data, a box indicates the interquartile range, and spikes extend to the upper and lower adjacent values, overlaid is the density.

The repeatability *h*^2^ of the agronomic and seed quality traits ranged for the winter trial from 0.26 to 0.96 and for the spring trial from 0.33 to 0.96. The AEM of the agronomic and seed quality traits were approximately normally distributed (Figures 1, 2). The proportion of the phenotypic variance of the agronomic and seed quality traits collected in the winter trial which was explained by population structure ranged from 0.00 to 0.79 (Table 1). For the winter trial, the average MRD (±standard error) of the inbreds of the WR-MCLUST group 1 vs. the inbreds of the WR-MCLUST group 2 was 0.45 (±0.01), whereas the average MRD of the inbreds of the winter trial vs. the inbreds of the spring trial was 0.31 (±0.01).

In the GWAS which was performed as a multiple forward regression analysis with 3910 SNPs, we observed 58 significant [*P* < 1.28e-05 (Bonferroni correction of α = 0.05)] SNP-trait associations for 12 of the 15 examined seed quality traits for the 184 *B. napus* inbreds of the spring trial. The SNP-seed quality trait associations explained individually from 0.0 to 63.1% of the phenotypic variance. For the 12 seed quality traits, between 1 and 21 SNPs were identified to be significantly associated with a single seed quality trait. These associations explained, on average, in a simultaneous fit 38.9% of the phenotypic variance for a single seed quality trait with a range from 11.7 to 87.2% (Table 2).

**Table 2:** **Single nucleotide polymorphism (SNP)-trait associations with ***P*** < 1.28e-05 (Bonferroni correction of α = 0.05) across the 184 inbreds of the spring trial**.

**Trait**	**Abbrevation**	**SNP array code**	**Chr.^a^**	**Position (bp)**	**Allele 1/2**	***P* value**	**Effect allele 1/2**	***P_*V*_*^b^ %**
BOF	BOF.A2.s.1	BN062891-0378	A2	4055363	C/T	4.49e-07	1.39	9.88
	BOF.A8.s.1	Bn-Scaffold000010-p1842065	A8	12587052	G/A	6.38e-08	−1.42	10.96
	BOF.C1.s.1	Bn-ctg7180014743505-p2383	C1	10885704	C/T	1.64e-06	−1.58	6.31
	BOF.C2.s.1	Bn-ctg7180014750900-p1843	C2	22964625	G/T	3.49e-07	−0.81	9.98
	BOF.s.1	Bn-Scaffold000037-p1731346			C/T	3.03e-07	1.71	12.16
	BOF.s.2	snp_BGA_3894			C/T	6.33e-09	1.87	18.66
	Simultaneous fit						41.85	
DBH	DBH.C0.s.1	Bn-ctg7180014764047-p127	C0	53561989	G/T	6.77e-06	−1.25	10.67
DTH	DTH.A1.s.1	Bn-Scaffold000042-p1923329	A1	1965029	C/T	3.11e-06	2.80	12.77
EMR	EMR.A7.s.1	UQ07A0010463	A7	917563	C/T	1.22e-05	0.89	11.17
EOF	EOF.A5.s.1	UQ11A0002096	A5	18017730	C/T	8.69e-07	−1.07	13.91
PTH	PTH.A3.s.1	Bn-Scaffold000005-p5062800	A3	4496358	C/T	7.78e-07	−40.26	13.41
	PTH.A9.s.1	Bn-Scaffold000022-p1083546	A9	11643646	C/T	2.22e-07	29.33	13.82
	Simultaneous fit						18.20	
ADF	ADF.A4.s.1	Bn-Scaffold000021-p1474068	A4	16611781	C/A	9.14e-06	−0.81	6.78
	ADF.A9.s.1	Bn-Scaffold000053-p842233	A9	31230574	G/A	2.94e-07	0.99	15.38
	ADF.C2.s.1	Bn-ctg7180014741828-p34349	C2	2353182	C/T	7.85e-10	−1.32	20.65
	ADF.C3.s.1	snp_BGA_201	C3	17957833	C/A	1.23e-06	0.69	7.73
	ADF.C7.s.1	Bn-ctg7180014766754-p1247	C7	986655	C/A	9.74e-06	−0.19	0.47
	ADF.C7.s.2	Bn-ctg7180014728682-p2037	C7	33844186	C/T	4.68e-06	0.47	1.04
	Simultaneous fit						38.19	
ADL	ADL.A2.s.1	Bn-Scaffold000062-p1221599	A2	157305	C/A	2.97e-09	−0.94	15.90
	ADL.A7.s.1	Bn-Scaffold000017-p2017197	A7	5709631	C/T	1.23e-05	0.87	13.60
	ADL.A9.s.1	Bn-ctg7180014758772-p913	A9	30359414	C/A	5.71e-06	−0.32	1.89
	ADL.C2.s.1	Bn-ctg7180014741828-p34349	C2	2353182	C/T	2.68e-10	−1.15	21.32
	ADL.C3.s.1	Bn-ctg7180014765519-p6291	C3	17808711	C/T	2.28e-06	0.55	1.43
	ADL.C3.s.2	snp_BGA_201	C3	17957833	C/A	7.20e-06	0.72	11.89
	ADL.C7.s.1	Bn-ctg7180014766754-p1247	C7	986655	C/A	1.32e-05	−0.13	0.31
	ADL.s.1	Bn-ctg7180014773771-p2060			C/A	3.91e-06	0.17	0.27
	Simultaneous fit						44.48	
CEL	CEL.C7.s.1	Bn-ctg7180014760120-p14495	C7	34977294	G/A	6.53e-07	−0.23	16.69
ERA	ERA.A2.s.1	Bn-ctg7180014766593-p2973	A2	4017247	G/A	7.60e-06	1.98	0.34
	ERA.A2.s.2	Bn-Scaffold000052-p168079	A2	20259210	G/A	6.43e-07	−7.60	3.59
	ERA.A7.s.1	Bn-Scaffold000017-p2107184	A7	5635674	C/T	2.89e-06	5.50	3.38
	ERA.A8.s.1	Bn-Scaffold000015-p2039258	A8	2396136	C/T	3.73e-06	−3.46	1.01
	ERA.A8.s.2	Bn-Scaffold000146-p254898	A8	7725352	C/T	8.02e-07	11.37	21.05
	ERA.A8.s.3	Bn-Scaffold000146-p168286	A8	7825612	C/T	4.97e-08	22.86	34.27
	ERA.A8.s.4	Bn-Scaffold000097-p464808	A8	10337576	C/T	3.51e-29	22.49	52.21
	ERA.A8.s.5	Bn-Scaffold000097-p271193	A8	10449583	G/T	9.85e-06	−18.79	42.37
	ERA.A9.s.1	Bn-ctg7180014738208-p2460	A9	2712534	C/T	1.31e-07	−7.11	4.26
	ERA.A9.s.2	Bn-Scaffold000121-p380018	A9	2739897	G/A	4.05e-06	−2.30	0.36
	ERA.A9.s.3	BN064849-0420	A9	22590517	C/T	4.23e-07	0.69	0.01
	ERA.C2.s.1	Bn-ctg7180014760667-p2108	C2	25150827	G/A	1.01e-07	6.03	3.86
	ERA.C3.s.1	Bn-ctg7180014766438-p3807	C3	5576905	G/A	1.27e-11	1.76	0.29
	ERA.C3.s.2	Bn-ctg7180014754298-p2473	C3	55022011	C/A	3.81e-06	0.14	0.00
	ERA.C3.s.3	Bn-ctg7180014745151-p4302	C3	55545323	C/T	1.69e-06	19.43	30.82
	ERA.C6.s.1	Bn-ctg7180014753577-p2215	C6	34010732	C/A	6.13e-08	7.76	5.56
	ERA.C8.s.1	Bn-ctg7180014765300-p3803	C8	31316701	G/A	2.51e-07	6.63	3.18
	ERA.A0.s.1	Bn-ctg7180014768425-p356	A0	6888575	G/A	5.32e-07	−13.06	27.20
	ERA.s.1	Bn-ctg7180014725700-p12794			C/A	1.02e-05	1.53	0.44
	ERA.s.2	Bn-ctg7180014770133-p1816			G/A	1.39e-07	−1.93	0.10
	ERA.s.3	Bn-Scaffold000038-p369909			G/A	1.11e-05	1.52	0.40
	Simultaneous fit						87.21	
GSL	GSL.A6.s.1	Bn-ctg7180014760121-p37899	A6	18128799	C/T	1.09e-05	7.05	1.06
	GSL.A9.s.1	Bn-Scaffold000006-p3146775	A9	3084578	C/T	1.93e-14	1.94	0.08
	GSL.A0.s.1	Bn-ctg7180014768425-p356	A0	6888575	G/A	1.95e-38	−51.78	63.10
	GSL.s.1	Bn-ctg7180014720122-p2129			C/T	6.51e-06	11.45	1.91
	Simultaneous fit						64.35	
HCL	HCL.A1.s.1	Bn-Scaffold000130-p478975	A1	18784468	C/T	3.48e-09	0.51	14.63
	HCL.A8.s.1	Bn-Scaffold000097-p464808	A8	10337576	C/T	4.52e-08	−0.40	14.71
	HCL.C7.s.1	Bn-ctg7180014760120-p14495	C7	34977294	G/A	3.54e-10	−0.42	25.61
	HCL.A0.s.1	Bn-Scaffold000099-p164187	A0	29091253	G/A	6.17e-09	−0.36	19.06
	Simultaneous fit						51.26	
LIA	LIA.A5.s.1	Bn-ctg7180014745444-p2596	A5	12864634	G/T	7.08e-06	0.70	12.46
MOI	MOI.C9.s.1	p5_5257_snp34	C9	19297345	G/A	4.90e-06	0.57	11.65
NDF	NDF.A7.s.1	Bn-Scaffold000017-p2017197	A7	5709631	C/T	1.76e-07	1.07	14.67
	NDF.C5.s.1	p6_3621_snp20	C5	41540882	G/T	9.59e-06	−0.47	2.56
	NDF.A0.s.1	Bn-ctg7180014768425-p356	A0	6888575	G/A	9.95e-11	−1.45	21.96
	Simultaneous fit						29.29	
OIL	OIL.C3.s.1	Bn-ctg7180014737168-p638	C3	513776	G/A	1.16e-07	1.78	15.77
	OIL.C5.s.1	Bn-ctg7180014702755-p1709	C5	9489169	C/A	4.41e-07	0.45	0.73
	Simultaneous fit						14.94	
OLA	OLA.A1.s.1	Bn-Scaffold000130-p478975	A1	18784468	C/T	2.92e-08	−3.85	10.49
	OLA.A6.s.1	Bn-ctg7180014756960-p1404	A6	18059898	G/T	4.90e-06	−1.51	1.34
	OLA.A8.s.1	Bn-Scaffold000097-p464808	A8	10337576	C/T	2.42e-09	3.77	19.36
	OLA.C3.s.1	Bn-ctg7180014765519-p6291	C3	17808711	C/T	4.06e-06	2.69	3.85
	Simultaneous fit						34.58	
SUL	SUL.A6.s.1	Bn-ctg7180014760121-p37899	A6	18128799	C/T	2.72e-06	0.03	0.85
	SUL.A9.s.1	Bn-Scaffold000006-p3146775	A9	3084578	C/T	5.90e-12	−0.01	0.03
	SUL.A0.s.1	Bn-ctg7180014768425-p356	A0	6888575	G/A	1.47e-36	−0.28	61.19
	Simultaneous fit						61.75	

*For abbreviations of the traits see Table 1*.

a *Chr. is the chromosome of the respective SNP; SNPs marked with a A0 or C0 could only be assigned to the genome of B. oleracea or B. rapa but not to a specific chromosome*.

b *P_V_ is the proportion of the explained phenotypic variance (%)*.

We observed for the 214 *B. napus* inbreds of the winter trial, 54 significant SNP associations for 12 seed quality traits (Table 3). We identified between 1 and 14 SNPs to be significantly [*P* < 1.28e-05 (Bonferroni correction of α = 0.05)] associated with a single seed quality trait. The identified loci explained individually from 0.0 to 67.3% of the phenotypic variance. The SNP-trait associations explained, on average, in a simultaneous fit 35.2% of the phenotypic variance for a single trait with a range of 9.8–76.7% (Table 3).

**Table 3:** **Single nucleotide polymorphism (SNP)-trait associations with ***P*** < 1.28e-05 (Bonferroni correction of α = 0.05) across the 214 inbreds of the winter trial**.

**Trait**	**Abbrevation**	**SNP array code**	**Chr.^a^**	**Position (bp)**	**Allele 1/2**	***P* value**	**Effect allele 1/2**	***P_*V*_*^b^ %**
BLC	BLC.A1.w.1	Bn-Scaffold000014-p848941	A1	4971489	C/T	3.61e-07	−0.25	11.54
	BLC.A9.w.1	Bn-Scaffold000053-p927209	A9	31325035	G/T	2.42e-06	0.46	27.72
	BLC.A9.w.2	Bn-Scaffold000077-p229981	A9	32873917	G/A	2.56e-18	−0.71	30.25
	BLC.C5.w.1	Bn-ctg7180011640898-p1781	C5	8100723	C/A	1.67e-07	−0.12	2.63
	Simultaneous fit						37.57	
BOF	BOF.A3.w.1	Bn-Scaffold000090-p1008061	A3	12037767	C/T	2.06e-06	1.04	14.67
	BOF.A0.w.1	Bn-Scaffold000002-p4747623	A0	43436688	C/A	2.02e-06	−0.92	5.93
	BOF.w.1	Bn-ctg7180014734592-p70			C/T	5.00e-11	−3.61	19.50
	BOF.w.1	Bn-Scaffold000032-p1987471			G/A	1.88e-06	0.00	0.00
	Simultaneous fit						34.04	
DAE	DAE.C2.w.1	Bn-ctg7180014740377-p6847	C2	8490346	G/A	3.00e-10	−2.63	17.48
DBH	DBH.A2.w.1	Bn-Scaffold000041-p1259335	A2	1420724	C/T	1.49e-06	−2.41	9.09
	DBH.A3.w.1	Bn-Scaffold000001-p1755819	A3	9705421	G/A	1.16e-06	1.06	7.00
	DBH.C2.w.1	Bn-ctg7180014734362-p2505	C2	43393890	C/T	4.00e-07	0.99	4.85
	DBH.C8.w.1	Bn-ctg7180014749298-p3202	C8	36366606	C/A	1.26e-05	0.06	0.02
	DBH.C8.w.2	BN075005-0426	C8	36713957	C/T	1.60e-06	−4.76	10.70
	DBH.w.1	Bn-ctg7180014758607-p5732			C/A	3.16e-08	1.25	15.53
	Simultaneous fit						37.89	
DTH	DTH.A9.w.1	Bn-ctg7180014738208-p2460	A9	2712534	C/T	5.72e-06	6.58	11.28
EMR	EMR.C2.w.1	Bn-ctg7180014740377-p6847	C2	8490346	G/A	5.30e-08	−2.41	13.80
	EMR.C5.w.1	snp_BGA_4916	C5	8326061	G/A	5.99e-13	2.63	28.40
	Simultaneous fit						28.76	
LOF	LOF.A7.w.1	Bn-Scaffold000012-p2678894	A7	10224576	G/T	9.45e-06	1.02	10.30
	LOF.C8.w.1	Bn-ctg7180014749298-p3202	C8	36366606	C/A	1.01e-06	0.02	0.00
	LOF.C8.w.2	Bn-ctg7180014732248-p707	C8	36668359	G/A	1.01e-05	−0.14	0.33
	LOF.C9.w.1	UQ03C0067042	C9	3443632	G/T	4.01e-06	0.13	0.28
	LOF.C9.w.2	Bn-ctg7180014727337-p703	C9	3705755	G/A	4.49e-06	1.52	11.34
	LOF.C0.w.1	Bn-ctg7180014738704-p1270	C0	56734079	C/T	6.45e-07	1.40	12.25
	Simultaneous fit						29.69	
LOM	LOM.A0.w.1	Bn-ctg7180014768425-p356	A0	6888575	G/A	1.12e-05	−1.08	9.34
MYD	MYD.A6.w.1	Bn-Scaffold000009-p1111712	A6	18877680	G/A	5.55e-06	−2.52	8.42
PTH	PTH.C5.w.1	Bn-ctg7180014734309-p3655	C5	42616932	G/T	8.48e-10	−17.55	15.77
	PTH.w.1	Bn-Scaffold000002-p1766964			G/A	1.14e-06	−3.28	2.07
	Simultaneous fit						16.00	
SAW	SAW.C5.w.1	Bn-ctg7180014771511-p3122	C5	38132173	C/T	3.76e-06	−0.57	9.50
	SAW.C7.w.1	Bn-ctg7180011792923-p2625	C7	39260815	C/A	1.24e-05	−0.13	0.09
	Simultaneous fit						10.47	
WIH	WIH.A1.w.1	Bn-Scaffold000033-p594082	A1	19725610	C/A	2.63e-06	1.65	6.99
	WIH.A5.w.1	Bn-Scaffold000075-p544803	A5	14988536	G/A	9.93e-06	0.16	0.20
	WIH.A7.w.1	Bn-Scaffold000003-p6314140	A7	19611453	G/A	1.85e-06	0.15	0.62
	WIH.A7.w.2	Bn-ctg7180014771687-p18821	A7	22478215	C/T	1.29e-07	1.53	13.34
	Simultaneous fit						21.74	
ADL	ADL.A9.w.1	Bn-Scaffold000393-p7477	A9	28209051	G/T	7.92e-07	3.63	11.29
AVA	AVA.A4.w.1	Bn-Scaffold000060-p374024	A4	7313760	G/A	4.14e-06	−0.34	9.76
CEL	CEL.A8.w.1	Bn-ctg7180014734032-p1283	A8	12795000	G/A	7.37e-07	−0.19	11.77
	CEL.w.1	Bn-ctg7180014725119-p15361			C/T	2.41e-06	0.07	1.19
	Simultaneous fit						12.52	
ERA	ERA.A8.w.1	Bn-Scaffold000015-p2264201	A8	2155967	G/A	3.20e-09	12.67	14.56
	ERA.A8.w.2	Bn-Scaffold000097-p710068	A8	10137532	C/A	4.27e-07	−15.03	16.10
	ERA.A8.w.3	Bn-ctg7180014771893-p599	A8	10225801	C/T	1.39e-08	−12.10	18.40
	ERA.A9.w.1	Bn-Scaffold000110-p349432	A9	2949845	G/A	1.20e-13	23.58	30.54
	ERA.C3.w.1	Bn-ctg7180014717095-p1564	C3	53048146	G/T	5.77e-25	22.79	39.38
	ERA.C3.w.2	Bn-ctg7180014745940-p4510	C3	54189048	C/A	6.25e-09	10.57	10.28
	ERA.C3.w.3	Bn-ctg7180014734187-p1715	C3	55135183	C/A	1.18e-12	6.63	4.34
	ERA.C3.w.4	Bn-ctg7180014745151-p4302	C3	55545323	C/T	1.66e-07	17.84	29.75
	Simultaneous fit						73.96	
GSL	GSL.A2.w.1	Bn-ctg7180014748062-p8451	A2	23876499	C/T	8.15e-54	−52.57	66.14
	GSL.A4.w.1	Bn-Scaffold000070-p872779	A4	10413384	G/A	8.74e-06	1.03	0.03
	GSL.A8.w.1	Bn-Scaffold000032-p328876	A8	9593875	G/A	5.64e-09	−0.68	0.01
	GSL.A9.w.1	Bn-Scaffold000051-p1490572	A9	2505543	C/T	1.11e-05	24.99	27.04
	GSL.A9.w.2	Bn-Scaffold000040-p186360	A9	2531260	G/A	2.27e-07	9.55	1.47
	GSL.A9.w.3	Bn-Scaffold000110-p573327	A9	2744611	C/T	5.29e-20	−4.74	0.55
	GSL.A9.w.4	BN049898-0393	A9	30354078	G/A	5.87e-06	0.53	0.00
	GSL.C1.w.1	p5_8563_snp7	C1	6390553	G/A	8.11e-07	9.46	0.43
	GSL.C1.w.2	Bn-ctg7180014746781-p3170	C1	6421746	G/A	4.99e-06	−8.77	0.47
	GSL.C5.w.1	Bn-ctg7180014774826-p5432	C5	12122482	C/T	1.15e-08	1.47	0.02
	GSL.C9.w.1	Bn-ctg7180014767584-p2156	C9	1664352	C/T	4.48e-07	−1.87	0.06
	GSL.C9.w.2	Bn-Scaffold000118-p574793	C9	1809481	C/A	5.32e-09	2.14	0.24
	GSL.w.1	Bn-Scaffold000092-p984593			G/A	8.45e-07	1.61	0.13
	GSL.w.2	Bn-Scaffold000094-p109812			G/A	4.26e-06	7.72	3.06
	Simultaneous fit						76.71	
HCL	HCL.A8.w.1	Bn-Scaffold000106-p682998	A8	1027180	G/A	1.28e-05	0.29	11.36
	HCL.A8.w.2	Bn-Scaffold000010-p3026578	A8	13719625	G/T	3.72e-07	0.39	16.83
	HCL.C8.w.1	Bn-ctg7180014732414-p9149	C8	25746465	G/A	1.25e-05	−0.37	3.85
	HCL.w.1	Bn-ctg7180014709967-p3714			G/A	2.52e-14	−0.44	25.82
	HCL.w.2	Bn-ctg7180014725119-p15361			C/T	1.04e-09	0.11	1.40
	HCL.w.3	Bn-Scaffold000031-p674411			C/T	9.09e-06	−0.09	0.15
	Simultaneous fit						41.22	
LIA	LIA.A7.w.1	Bn-Scaffold000018-p869005	A7	278027	G/A	9.28e-06	0.81	12.16
	LIA.A8.w.1	Bn-Scaffold000097-p710068	A8	10137532	C/A	7.63e-06	1.44	18.55
	LIA.A9.w.1	Bn-Scaffold000110-p349432	A9	2949845	G/A	2.52e-07	−1.47	24.65
	LIA.C2.w.1	Bn-ctg7180014733329-p2936	C2	44856112	C/T	5.22e-06	−0.80	6.43
	LIA.C3.w.1	Bn-ctg7180014726380-p989	C3	5337555	C/A	5.96e-17	1.39	28.96
	Simultaneous fit						43.87	
NDF	NDF.C2.w.1	Bn-ctg7180014746332-p7435	C2	45024709	G/T	1.03e-07	0.72	14.33
	NDF.C3.w.1	Bn-Scaffold000032-p835836	C3	53374601	G/T	6.60e-06	−0.13	0.36
	Simultaneous fit						14.60	
OIL	OIL.A1.w.1	Bn-Scaffold000011-p1364245	A1	2717777	G/A	1.05e-05	−1.57	5.06
	OIL.C3.w.1	Bn-ctg7180014717095-p1564	C3	53048146	G/T	5.02e-07	1.50	11.70
	Simultaneous fit						20.21	
OLA	OLA.A9.w.1	Bn-Scaffold000110-p349432	A9	2949845	G/A	1.98e-20	5.31	30.93
	OLA.A9.w.2	BN049898-0393	A9	30354078	G/A	3.04e-06	0.59	0.15
	OLA.A9.w.3	Bn-ctg7180014758772-p913	A9	30359414	C/A	4.35e-06	−0.41	0.11
	OLA.C3.w.1	Bn-ctg7180014717095-p2357	C3	53047354	C/T	8.18e-08	4.40	19.63
	OLA.C8.w.1	Bn-ctg7180014732414-p9149	C8	25746465	G/A	5.87e-06	1.58	0.77
	OLA.A0.w.1	Bn-Scaffold000010-p2545490	A0	37536253	G/T	1.60e-08	4.62	24.24
	OLA.w.1	Bn-ctg7180014709374-p768			C/T	4.72e-06	0.82	0.61
	OLA.w.2	Bn-ctg7180014709661-p1084			C/A	2.83e-07	1.62	0.87
	OLA.w.3	Bn-ctg7180014724744-p69			G/A	6.64e-06	0.83	1.41
	Simultaneous fit						39.86	
PRT	PRT.C6.w.1	Bn-ctg7180014756759-p1575	C6	1625464	G/T	1.69e-06	1.03	10.76
SUL	SUL.A2.w.1	Bn-ctg7180014748062-p8451	A2	23876499	C/T	1.93e-52	−0.34	67.34
	SUL.A9.w.1	Bn-Scaffold000040-p186360	A9	2531260	G/A	3.68e-06	0.01	0.04
	SUL.A9.w.2	Bn-Scaffold000110-p573327	A9	2744611	C/T	3.49e-16	0.00	0.00
	Simultaneous fit						68.06	

*For abbreviations of the traits see Table 1*.

a *Chr. is the chromosome of the respective SNP; SNPs marked with a A0 or C0 could only be assigned to the genome of B. oleracea or B. rapa but not to a specific chromosome*.

b *P_V_ is the proportion of the explained phenotypic variance (%)*.

For the association analysis of the agronomic traits, we observed for the inbreds of the spring trial 12 SNP-trait associations for six of the 15 agronomic traits with a *P* < 1.28e-05 (Table 2). These significant associations explained individually from 6.3 to 18.7% of the phenotypic variance. Furthermore, for these traits, we found 1–6 SNP-trait associations which explained, on average, in a simultaneous fit 18.1% of the phenotypic variance (Table 2).

For the winter trial, we found 34 significant SNP-trait associations for 12 of the 15 agronomic traits (Table 3) and they explained individually from 0.0 to 30.2% of the phenotypic variance. We observed 1–6 significant SNPs to be associated with a trait and they explained on average in a simultaneous fit 21.9% (range 8.4–37.9%) of the phenotypic variance (Table 3).

For the seed quality trait ERA a co-localized SNP association between the spring and the winter trial could be identified on chromosome C3, whereas no associated SNP co-localizations between the spring and the winter trial were examined for the agronomic traits (Tables 2, 3).

In a BLAST search within a distance of 2.5 Mbp around the SNP-trait associations, 28 hits of potential candidate genes with a BLAST-score of ≥100 and a sequence identity of ≥70% to *A. thaliana* or *B. rapa* could be found for the agronomic SNP-trait associations of the inbreds of the spring trial and 34 candidate gene hits for the inbreds of the winter trial. Furthermore, for the seed quality SNP-trait associations 82 candidate gene hits were identified for the inbreds of the spring trial and 105 candidate gene hits for the inbreds of the winter trial (Tables 4, 5, Tables S1–S6).

**Table 4:** **BLAST search results for pre-selected candidate genes for the single nucleotide polymorphism (SNP)-trait associations with ***P*** < 1.28e-05 (Bonferroni correction of α = 0.05) within a distance of 2.5 Mbp around the SNP-trait associations across the 184 inbreds of the spring trial**.

**Trait**	**SNP abbrevation**	**Chr.^a^**	**SNP position (bp)**	**Candidate gene**	**NCBI gene ID**	**Locus tag**	**Identity (%)**	**Start position**	**End position**	**Gene position**	**Distance to SNP**
**AGRONOMIC TRAITS**											
BOF	BOF.A2.s.1	A02	4055363	GNC	835788	AT5G56860	79	4162702	4163847	4163275	107912
				VIN3	835844	AT5G57380	80	3864072	3864747	3864410	190954
				FT	842859	AT1G65480	87	6375965	6376243	6376104	2320741
	BOF.A8.s.1	A08	12587052	FD	829744	AT4G35900	76	12446708	12448076	12447392	139660
				AP1	843244	AT1G69120	84	15078189	15078309	15078249	2491197
				SOC1/AGL20	819174	AT2G45660	78	15081763	15081902	15081833	2494781
	BOF.C1.s.1	C01	10885704	SVP	816787	AT2G22540	78	11245513	11245696	11245605	359901
				AGL24	828556	AT4G24540	77	11245357	11246128	11245743	360039
				SOC1/AGL20	819174	AT2G45660	83	9682339	9682526	9682433	1203272
				GNC	835788	AT5G56860	84	12641365	12641490	12641428	1755724
	BOF.C2.s.1	C02	22964625	TPS1	844194	AT1G78580	93	22384412	22384513	22384463	580163
				LD	827904	AT4G02560	90	24966748	24966982	24966865	2002240
				FT	842859	AT1G65480	93	20908311	20908383	20908347	2056278
EOF	EOF.A5.s.1	A05	18017730	SPA3	820767	AT3G15354	77	18186337	18189758	18188048	170318
				AP1	843244	AT1G69120	84	17789288	17789416	17789352	228378
				VRN1	821432	AT3G18990	82	16379258	16379735	16379497	1638234
**SEED QUALITY TRAITS**											
ADF	ADF.A4.s.1	A04	16611781	GAUT7	818447	AT2G38650	81	16723369	16725115	16724242	112461
	ADF.C7.s.1	C07	986655	GAUT10	816611	AT2G20810	85	342567	342660	342614	644042
ADL	ADL.A7.s.1	A07	5709631	QUA1	822105	AT3G25140	87	5464119	5465842	5464981	244651
	ADL.C7.s.1	C07	986655	GAUT10	816611	AT2G20810	85	342567	342660	342614	644042
ERA	ERA.A8.s.3	A08	7825612	FAE1/KCS18	829603	AT4G34520	84	10187612	10189220	10188416	2362804
	ERA.A8.s.4	A08	10337576	FAE1/KCS18	829603	AT4G34520	84	10187612	10189220	10188416	149160
	ERA.C3.s.3	C03	55545323	FAE1/KCS18	829603	AT4G34520	84	55684172	55685778	55684975	139652
GSL	GSL.A6.s.1	A06	18128799	SUR1-like	103837982	LOC103837982	79	17341006	17341137	17341072	787728
				OBP2	837277	AT1G07640	84	17014601	17014756	17014679	1114121
	GSL.A9.s.1	A09	3084578	ATR1/MYB34	836210	AT5G60890	77	2698822	2699466	2699144	385434
				OBP2	837277	AT1G07640	80	2683543	2683694	2683619	400960
				SUR1-like	103837982	LOC103837982	99	5083167	5085820	5084494	1999916
				SUR1	816585	AT2G20610	86	5085356	5085820	5085588	2001010
				AOP1	828100	AT4G03070	82	697138	697534	697336	2387242
				AOP2	828102	AT4G03060	79	692952	693321	693137	2391442
				AOP3	828104	AT4G03050	79	692952	693321	693137	2391442
HCL	HCL.A1.s.1	A01	18784468	GAUT1	825285	AT3G61130	91	20219967	20220082	20220025	1435557
NDF	NDF.A7.s.1	A07	5709631	QUA1	822105	AT3G25140	87	5464119	5465842	5464981	244651
				GAUT10	816611	AT2G20810	83	4127749	4129555	4128652	1580979
OLA	OLA.A1.s.1	A01	18784468	FAR7	832303	AT5G22420	78	21097835	21098001	21097918	2313450
	OLA.A6.s.1	A06	18059898	FAR7	832303	AT5G22420	76	15755098	15755262	15755180	2304718
	OLA.A8.s.1	A08	10337576	CER4	829521	AT4G33790	92	10388255	10388380	10388318	50742
				FAR1	832311	AT5G22500	79	10389865	10390018	10389942	52366
				FAR7	832303	AT5G22420	79	11667520	11667667	11667594	1330018
	OLA.C3.s.1	C03	17808711	FAR7	832303	AT5G22420	78	16964614	16964759	16964687	844025
SUL	SUL.A6.s.1	A06	18128799	ATSERAT3;1	816271	AT2G17640	76	17771333	17772947	17772140	356659
				APK3	821077	AT3G03900	76	17301192	17301351	17301272	827528
				APK4	836888	AT5G67520	76	17300756	17301552	17301154	827645
	SUL.A9.s.1	A09	3084578	APK4	836888	AT5G67520	80	3615223	3615740	3615482	530904
				APK3	821077	AT3G03900	78	3615562	3615705	3615634	531056
				ATSERAT3;1	816271	AT2G17640	75	4588363	4588772	4588568	1503990

*For abbreviations of the traits see Table 1 and for the full list of candidate genes see Table S1*.

a *Chr. is the chromosome of the respective SNP*.

**Table 5:** **BLAST search results for pre-selected candidate genes for the single nucleotide polymorphism (SNP)-trait associations with ***P*** < 1.28e-05 (Bonferroni correction of α = 0.05) within a distance of 2.5 Mbp around the SNP-trait associations across the 214 inbreds of the winter trial**.

**Trait**	**SNP abbrevation**	**Chr.^a^**	**SNP position (bp)**	**Candidate gene**	**NCBI gene ID**	**Locus tag**	**Identity (%)**	**Start position**	**End position**	**Gene position**	**Distance to SNP**
**AGRONIMIC TRAITS**											
BLC	BLC.A1.w.1	A01	4971489	TT8	826571	AT4G09820	79	5634140	5634445	5634293	662804
BOF	BOF.A3.w.1	A03	12037767	SPA2	826712	AT4G11110	85	11828002	11828089	11828046	209722
				VRN2	827392	AT4G16845	83	12669404	12669541	12669473	631706
				GA1	828182	AT4G02780	88	12712210	12712448	12712329	674562
				LD	827904	AT4G02560	80	12787525	12787656	12787591	749824
DBH	DBH.A2.w.1	A02	1420724	AT4G36140	829771	AT4G36140	75	1747205	1747359	1747282	326558
				AT5G17970	831664	AT5G17970	87	1747079	1747554	1747317	326593
				TAO1	834478	AT5G44510	75	1833113	1833324	1833219	412495
				AT5G18350	831953	AT5G18350	81	1833640	1833848	1833744	413020
	DBH.A3.w.1	A03	9705421	MYB12	819359	AT2G47460	75	10295645	10296753	10296199	590778
				EXPA1	843288	AT1G69530	79	9045377	9045610	9045494	659928
	DBH.C2.w.1	C02	43393890	MYB12	819359	AT2G47460	84	41992882	41992974	41992928	1400962
	DBH.C8.w.2	C08	36713957	AT1G12280	837782	AT1G12280	81	36207725	36208740	36208233	505725
**SEED QUALITY TRAITS**											
ADL	ADL.A9.w.1	A09	28209051	GAUT1	825285	AT3G61130	81	27661029	27663693	27662361	546690
ERA	ERA.A8.w.3	A08	10225801	FAE1/KCS18	829603	AT4G34520	84	10187612	10189220	10188416	37385
	ERA.C3.w.4	C03	55545323	FAE1/KCS18	829603	AT4G34520	84	55684172	55685778	55684975	139652
GSL	GSL.A2.w.1	A02	23876499	MAM3/IMS2	832366	AT5G23020	80	23671927	23672445	23672186	204313
				MAM1	832365	AT5G23010	83	23671132	23671429	23671281	205219
				SUR1-like	103837982	LOC103837982	78	22869827	22870204	22870016	1006484
	GSL.A4.w.1	A04	10413384	CYP79B3	816765	AT2G22330	89	10803199	10804213	10803706	390322
				CYP79B2	830154	AT4G39950	83	10804805	10805443	10805124	391740
				SUR1-like	103837982	LOC103837982	81	10808649	10808789	10808719	395335
	GSL.A8.w.1	A08	9593875	GSH1	828409	AT4G23100	92	9811847	9811945	9811896	218021
				SUR1-like	103837982	LOC103837982	79	11574819	11574955	11574887	1981012
	GSL.A9.w.1	A09	2505543	OBP2	837277	AT1G07640	80	2683543	2683694	2683619	178076
				ATR1/MYB34	836210	AT5G60890	77	2698822	2699466	2699144	193601
				SUR1-like	103837982	LOC103837982	78	1012252	1012504	1012378	1493165
				AOP1	828100	AT4G03070	82	697138	697534	697336	1808207
				AOP2	828102	AT4G03060	79	692952	693321	693137	1812407
				AOP3	828104	AT4G03050	79	692952	693321	693137	1812407
	GSL.A9.w.4	A09	30354078	HAG1/MYB28	836263	AT5G61420	84	30597664	30597759	30597712	243634
				HIG2/MYB122	843748	AT1G74080	76	30597950	30598349	30598150	244072
				HIG1/MYB51	838438	AT1G18570	81	30597946	30598603	30598275	244197
				SUR1-like	103837982	LOC103837982	86	31250926	31251030	31250978	896900
	GSL.C1.w.2	C01	6421746	SUR1	816585	AT2G20610	81	5909358	5909522	5909440	512306
				SUR1-like	103837982	LOC103837982	82	5908889	5909046	5908968	512779
	GSL.C5.w.1	C05	12122482	SUR1-like	103837982	LOC103837982	79	11212898	11213167	11213033	909450
				OBP2	837277	AT1G07640	79	10373511	10373656	10373584	1748899
	GSL.C9.w.2	C09	1809481	OBP2	837277	AT1G07640	80	2905001	2905152	2905077	1095596
				ATR1/MYB34	836210	AT5G60890	77	2926750	2927390	2927070	1117589
				HAG1/MYB28	836263	AT5G61420	80	3099297	3100168	3099733	1290252
				HIG1/MYB51	838438	AT1G18570	83	3100044	3100138	3100091	1290610
				HIG2/MYB122	843748	AT1G74080	79	3100045	3100165	3100105	1290624
				AOP1	828100	AT4G03070	82	231456	231860	231658	1577823
				AOP2	828102	AT4G03060	80	216661	217030	216846	1592636
				AOP3	828104	AT4G03050	80	216661	217030	216846	1592636
				SUR1-like	103837982	LOC103837982	77	3789954	3790327	3790141	1980660
HCL	HCL.A8.w.2	A08	13719625	GAUT12	835558	AT5G54690	88	14184821	14185072	14184947	465322
NDF	NDF.C3.w.1	C03	53374601	GAUT10	816611	AT2G20810	78	52377904	52378115	52378010	996592
OLA	OLA.A9.w.1	A09	2949845	FAR7	832303	AT5G22420	79	4862541	4862688	4862615	1912770
	OLA.A9.w.2	A09	30354078	FAR7	832303	AT5G22420	79	31423271	31423418	31423345	1069267
	OLA.C3.w.1	C03	53047354	FAR7	832303	AT5G22420	77	53458768	53458914	53458841	411487
	OLA.C8.w.1	C08	25746465	FAR7	832303	AT5G22420	77	25625877	25626022	25625950	120516
SUL	SUL.A9.w.1	A09	2531260	APK4	836888	AT5G67520	80	3615223	3615740	3615482	1084222
				APK3	821077	AT3G03900	78	3615562	3615705	3615634	1084374
				ATSERAT3;1	816271	AT2G17640	75	4588363	4588772	4588568	2057308

*For abbreviations of the traits see Table 1 and for the full list of candidate genes see Table S2*.

a *Chr. is the chromosome of the respective SNP*.

## 4. Discussion

In our *B. napus* diversity set, the nonlinear trend line of the LD measure *r*^2^ decayed below the significance threshold within a distance of 677 kb. Bus et al. (2011) estimated based on 89 SSR markers that the pairwise LD within our *B. napus* diversity set decayed within a genetic map distance of ~1 cM. This corresponds to about 500 kb (Arumuganathan and Earle, 1991) and is in good accordance to the value observed in our study. Furthermore, the LD observed by Delourme et al. (2013) in a *B. napus* collection of 313 inbred lines decayed within 0.6–0.7 cM (~300–350 kb) for their whole collection and within 1.2 cM for their 00İ winter types. The extent of LD in the collection of Delourme et al. (2013) varied depending on the linkage group and the collection between 0.2 and 3.4 cM (~0.1–1.7 Mbp). In addition, Qian et al. (2014) identified in the allopolyploid *B. napus* genome on average an around ten times more rapidly decayed mean LD for the A-genome (0.25–0.30 Mbp) than for the C-genome (2.00–2.50 Mbp). Due to the variation within the decay of LD between *B. napus* subgroups and even between chromosomes, potential candidate genes for SNP-trait associations were searched in our study 2.5 Mbp up- and downstream of each association.

In our study, 1577 SNPs mapped to the A genome, whereas 1889 SNPs mapped to the C genome of *B. napus* which is on average one SNP every 0.7 cM expecting that the *B. napus* genome has a length of ~2500 cM (Ecke et al., 2010; Delourme et al., 2013). As the pairwise LD within our *B. napus* diversity set decayed within a genetic map distance of ~1 cM (677 kb), a total of 96.8% of the adjacent SNPs on the A genome and 83.0% of the adjacent SNPs on the C genome had a distance smaller than the average range of LD. These results indicate that the SNP marker density of our study is expected to provide a sufficient power to detect SNP-trait associations in the *B. napus* diversity set.

SNP-trait associations detected for the agronomic traits in the spring and winter trial explained in a simultaneous fit on average 18.1 and 21.9% of the phenotypic variance, respectively (Tables 2, 3). This is in accordance with the results of Mei et al. (2009) who observed, on average, an explained phenotypic variance for flowering time and plant height of 16.4% in a QTL analysis based on a segregating population.

The SNP-trait associations for the seed quality traits in the spring and winter trial explained in a simultaneous fit on average 38.9 and 35.2% of the phenotypic variance, respectively (Tables 2, 3). These values were much higher than those observed for the agronomic traits which indicates that the latter are genetically more complex inherited than the rather mono- or oligogenic seed quality traits.

**Figure 3:**
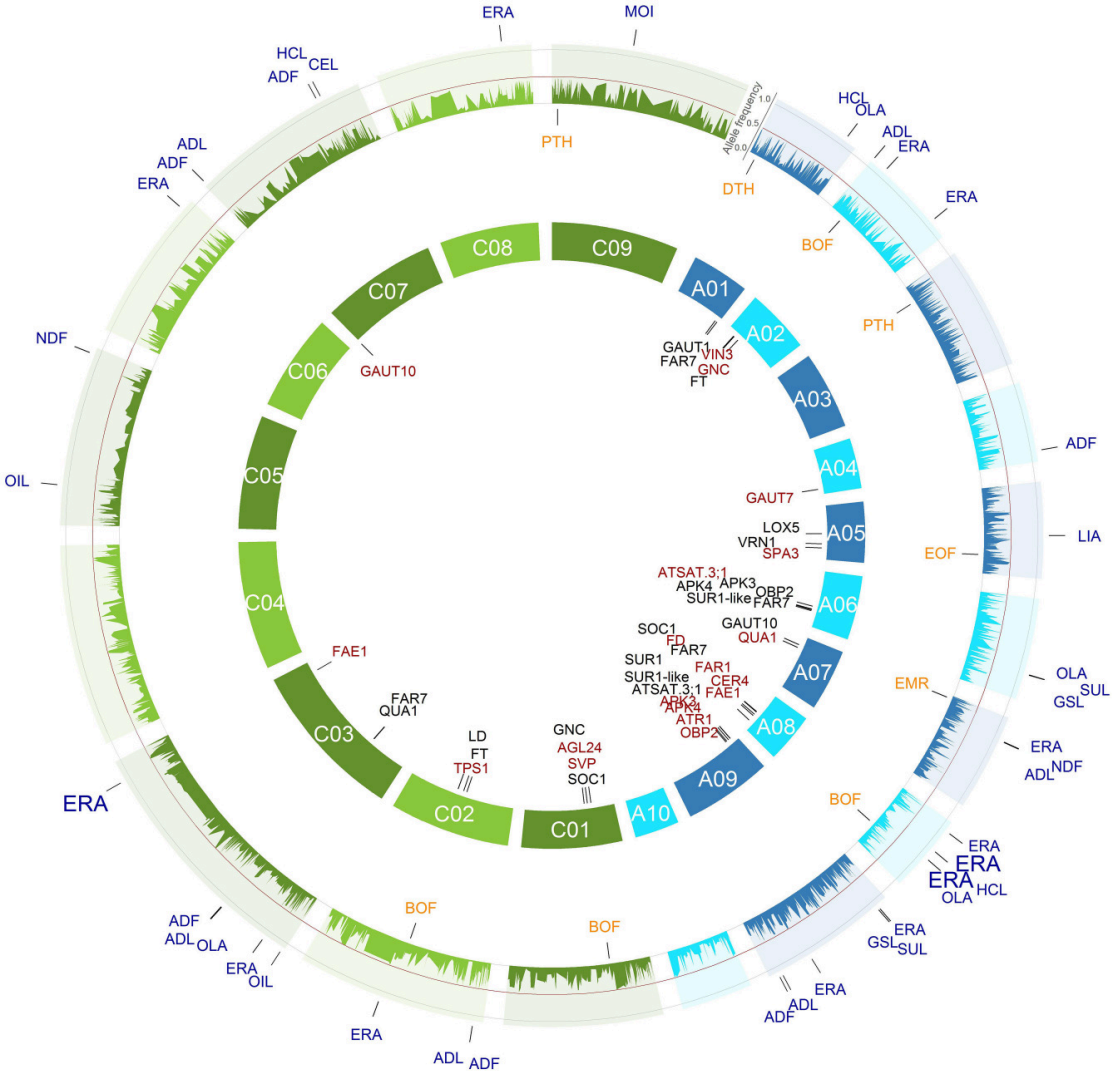
**All single nucleotide polymorphism (SNP)-trait associations with a ***P*** < 1.28e-05 (Bonferroni correction of α = 0.05) identified across the 184 inbreds of the spring trial and their respective positions are marked on the ***B. napus*** genome**. The 3466 SNPs with their minor allele frequencies in the spring trial are given in the outer circle. The SNPs associated with the agronomic SNP-trait associations are plotted in orange below the allele frequency circle and the seed quality SNP-trait associations in blue outside the allele frequency circle. The size of the letters is related to the proportion of the variance explained by the associations. In the inner circle of the 19 chromosomes, the candidate genes were plotted to their mapping position on the *B. napus* reference genome. The A genome is colored blue and the C genome green.

**Figure 4:**
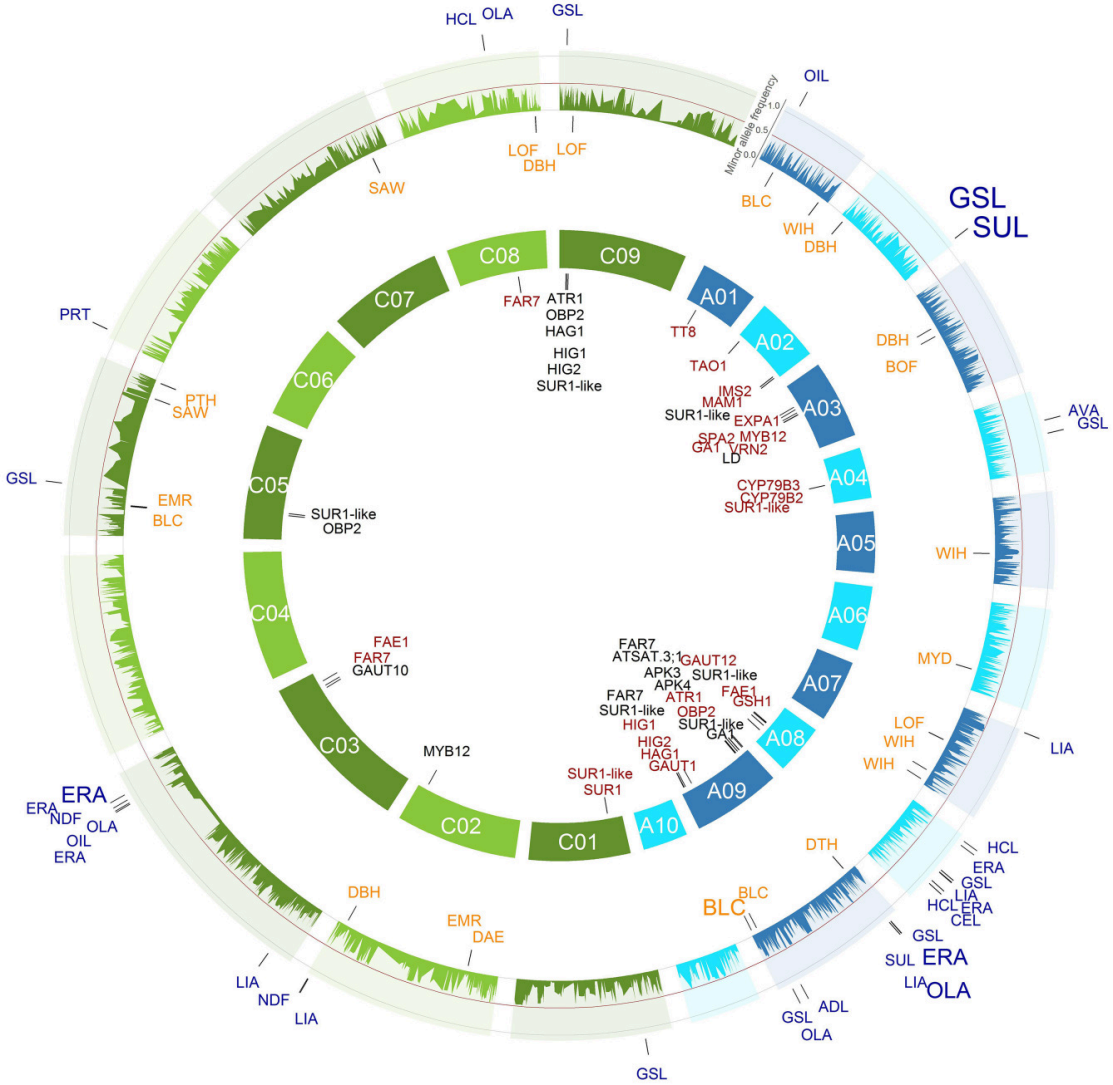
**All single nucleotide polymorphism (SNP)-trait associations with a ***P*** < 1.28e-05 (Bonferroni correction of α = 0.05) identified across the 214 inbreds of the winter trial and their respective positions are marked on the ***B. napus*** genome**. The 3466 SNPs with their minor allele frequencies in the winter trial are given in the outer circle. The SNPs associated with the agronomic SNP-trait associations are plotted in orange below the allele frequency circle and the seed quality SNP-trait associations in blue outside the allele frequency circle. The size of the letters is related to the proportion of the variance explained by the associations. In the inner circle of the 19 chromosomes, the candidate genes were plotted to their mapping position on the *B. napus* reference genome. The A genome is colored blue and the C genome green.

For most of the examined agronomic and seed quality traits, a couple of major SNP-trait associations with a valuable percentage of explained phenotypic variance were identified which could be useful for MAS in *B. napus* (Tables 2, 3, Figures 3, 4, Tables S1, S2, Figures S3, S4).

### 4.1. Genome-wide associations of agronomic traits

#### 4.1.1. Begin of flowering (BOF) and end of flowering (EOF)

In *B. napus* breeding, flowering time adaptation is one of the breeding goal (Wang et al., 2011a). For example, spring types flower early without vernalization to utilize fully the short vegetation period in regions with strong winters. Therefore, alleles which have a low frequency in a germplasm type and a desirable effect on the trait of interest could be selected for to improve this trait. The allele one at the SNP-BOF associations BOF.A8.s.1, BOF.C1.s.1, and BOF.C2.s.1 leads to an early flowering and occurs with a frequency of 84.3, 5.4, and 37.3% in the spring OSR cultivars (Figure 3, Table 2). According to this, these associations except for BOF.A8.s.1 at which the favorable allele has already a frequency of 47.8%, could also be used for MAS to also improve early flowering in the winter OSR.

High temperatures at flowering reduce yield of *B. napus* (Angadi et al., 2000). Thus, with climate change, high temperatures at flowering are expected to occur more often. Therefore, it could be advantageous for some geographic regions to breed early flowering winter OSR cultivars. The early flowering alleles of BOF.C1.s.1, and BOF.C2.s.1 which have low frequencies (1.6 and 3.8%) in the winter OSR cultivars might be interesting for breeding early flowering cultivars. In contrast, the allele which causes late flowering at BOF.A2.s.1 is a major allele in the spring and winter OSR but not present in the semi-winter OSR. From this it follows that the early flowering allele in the semi-winter OSR might be a potential target for MAS of early flowering cultivars in the spring and winter OSR.

The SNP-BOF associations BOF.A2.s.1 and BOF.A3.w.1 co-located with the QTL *dtf2.1* and *dtf2.3* as well as *dtf3.1* - *dtf3.4* identified by Udall et al. (2006) and Quijada et al. (2006) in a *B. napus* DH population and its testcross progeny. In addition, Wang et al. (2011a) identified a major flowering time QTL on chromosome A3 at 49.8 cM which co-localized with a putative rapeseed ortholog of *FRIGIDA*. Furthermore, Quijada et al. (2006) also identified the QTL *dtf12b* at 33.1 and 36.7 cM which is in good accordance with our BOF.C2.1 association. The validation of genome regions in several experiments with different environmental conditions as well as different genetic background suggests that these regions have a major impact on the trait of interest.

Several known genes which are related to flowering could be localized within a distance of up to 2.5 Mbp to the associations for BOF identified in this study (Tables 4, 5). Only 580 kb downstream of BOF.C2.s.1 the TREHALOSE-6-PHOSPHATE SYNTHASE 1 (*TPS1*) which causes in case of a loss *A. thaliana* to flower extremely late could be identified (Wahl et al., 2013). The FLOWERING LOCUS T gene (*FT*) which is antagonistic with its homologous gene, TERMINAL FLOWER1 (*TFL1*) and promotes flowering together with the gene *LFY* could be mapped 2.3 Mbp upstream of BOF.A2.s.1 and 2.1 Mbp downstream of BOF.C2.s.1. Furthermore, the gene *GNC* which is a GATA transcription factor act upstream from the flowering time regulator SUPPRESSOR OF OVEREXPRESSION OF CONSTANS1 (*SOC1*/*AGL20*) to directly repress *SOC1* expression and thereby repress flowering (Richter et al., 2013) and could be located only 108 kb upstream of BOF.A2.s.1 as well as 1.8 Mbp upstream of BOF.C1.s.1. In addition, 360 kb upstream of BOF.C1.s.1 the MADS-box gene AGAMOUS-LIKE 24 (*AGL24*) which promotes flowering by a positive-feedback loop with *SOC1* at the shoot apex (Liu et al., 2008) could be mapped. The gene *FD* first identified by Koornneef et al. (1991) in *A. thaliana* could be mapped within a distance of 140 kb downstream of the BOF.A8.s.1. The *FD* gene activates in a complex with the *FLOWERING LOCUS T* (*FT*) protein so-called floral identity genes such as *APETALA1* (*AP1*) (Wigge et al., 2005) which could be mapped to BOF.A8.s.1 and EOF.A5.s.1. The VERNALIZATION2 (*VRN2*) gene stably maintains FLOWERING LOCUS C (*FLC*) repression after a cold treatment (Gendall et al., 2001) and could be mapped to BOF.A3.w.1 for the inbreds of the winter trial. Further research is needed to examine which are the causal polymorphisms in these genome regions.

For the trait end of flowering (EOF), we identified one significant SNP-EOF association EOF.A5.s.1 which explained 13.9% of the phenotypic variance (Figure 3, Table 2). The late ending of flowering allele is already present in 67.2 and 85.7% of the spring and winter OSR cultivars in this study. The low number of EOF-SNP associations might be due to the fact that this trait was evaluated at only four locations in contrast to the trait BOF which has been assessed at seven locations.

#### 4.1.2. Plant height (PTH)

The significant SNP-PTH association PTH.A3.s.1 was located in the same genome region as the QTL *ph3.3* for plant height which was detected in a DH population on chromosome A3 at 36.5 cM (Udall et al., 2006) (Figure 3, Table 2). If PTH.A3.s.1 and ph3.3 characterize the same locus, however, requires further research.

The SNP-PTH association PTH.A3.s.1 caused a reduction in plant height and is present in 93.1% of the spring OSR but only present in 46.2% of the winter OSR. Furthermore, the allele which reduce the plant height at the locus PTH.A9.s.1 is only present in 4.4 and 11.5% of the spring and winter OSR, respectively. From this it follows that both SNP-PTH associations could be useful to reduce the plant height in *B. napus* winter OSR variaties.

#### 4.1.3. Disease status before harvest (DBH)

The trait DBH is a summary score and included various diseases before harvest, such as *Alternaria brassicae, Sclerotinia sclerotiorum*, and *Leptosphaeria maculans*. The SNP-DBH associations DBH.A2.w.1 and DBH.A3.w.1 were in good accordance with *S. sclerotiorum* resistance QTL on chromosome A2 at 11.0 cM and on chromosome A3 at 68.0 cM detected by Zhao et al. (2006) (Figure 4, Table 3).

The first allele of DBH.A2.w.1 which is present in 2.7% of the winter OSR and 6.9% of the spring OSR is responsible for an improved disease status, whereas the allele of DBH.A3.w.1 which causes the improved disease status is already present in 88.0% of the winter OSR and 40.2% of the spring OSR. Also the resistance allele of DBH.C8.w.2 is already present in most of the modern cultivars. Thus, DBH.A2.w.1 might be a promising candidate for MAS to increase the disease status in winter OSR as well as spring OSR.

#### 4.1.4. Yield (DTH)

We found the two significant association DTH.A1.s.1 and DTH.A9.w.1 and the first alleles caused an increase in seed yield between 2.8 and 6.6% (Figures 3, 4, Tables 2, 3). However, the low number of significant SNP associations for this quantitative and highly complex trait is most likely due to the fact that the trait was examined at only two locations. This suggests that the SNP-DTH associations are not directly usable for MAS.

### 4.2. Genome-wide associations of seed quality traits

#### 4.2.1. Seed oil content (OIL)

*B. napus* is planted for oil production and, therefore, an maximization of oil content in the seeds is a major goal in the breeding process (Zhao et al., 2007; Würschum et al., 2012). In our study, four significant SNP-OIL associations were detected on the chromosomes A1, C3, and C5 for the inbreds of the spring and winter trial (Figures 3, 4, Tables 2, 3). The position of the identified SNP-OIL association OIL.C3.w.1 was in accordance with that of the QTL detected by Qiu et al. (2006) on the chromosome C3 at 88.9 and 89.7 cM using a TNDH population. This result validates the OIL.C3.w.1 association and indicates that this genome region seems to be of particular importance.

The allele which is responsible for an increase of oil content is present in most of the spring and winter OSR for the associations OIL.A1.w.1 and OIL.C3.s.1, whereas it is only present in same of the spring and winter OSR for the associations OIL.C3.w.1 and OIL.C5.s.1 and, thus, provides an opportunity for MAS.

#### 4.2.2. Erucic acid concentration (ERA; C22:1)

We detected several significant SNP-ERA associations which explained in a simultaneous fit for the spring trial, the winter trial, and the WR-MCLUST group 1 with 87.2, 74.0, and 80.5% a large proportion of the phenotypic variance, respectively (Figures 3, 4, Tables 2, 3). Our findings are in good accordance with the results of QTL for erucic acid concentration of previous studies (Barret et al., 1998; Fourmann et al., 1998; Burns et al., 2003; Qiu et al., 2006; Basunanda et al., 2007; Zhao et al., 2007; Smooker et al., 2011). This supports that the diversity set used in our study is a powerful tool to dissect quantitative traits.

The mapping positions of the major SNP-ERA associations which were observed for the summer trial were close by or in some cases even identical to that observed for the winter trial (Tables 2, 3, Table S3). These results are in accordance with the breeding history that the low erucic acid variation in the winter OSR has been introduced from the spring cultivar “Liho” (Friedt and Snowdon, 2010).

Barret et al. (1998) isolated two α*-ketoacyl-CoA synthase* sequences from a *B. napus* immature embryocDNAlibrary. This enzyme controls erucic acid synthesis in *B. napus* seeds and was first described in *A. thaliana* where it is encoded by the *FATTY ACID ELONGATION1* (*FAE1* or *KCS18*) gene (James and Dooner, 1990; James et al., 1995). Using a BLAST search, we could map this gene of *A. thaliana* very closely to the major SNP-ERA associations on the chromosomes A8, A9, and C3 (Tables 4, 5). This finding is in accordance with results of Barret et al. (1998) who already localized these *FAE1* genes to the loci E1 and E2 on the chromosomes A8 and C3 which were already known to be tightly linked to erucic acid content (Jourdren et al., 1996). Qiu et al. (2006), Basunanda et al. (2007), and Smooker et al. (2011) could specify these positions on the chromosome A8 and C3 by QTL analyses. Li et al. (2014) identified the two associations with erucic acid content on chromosome A8 at 9.5 Mbp and C3 at 63.7 Mbp within a distance of 233 and 128 kb away from the genes *BnaFAE1.1* and *BnaFAE1.2*, respectively. Thus, our examined SNP-ERA associations on chromosome A8 at 10.3 Mbp and on chromosome C3 at 55.5 Mbp were located in the same genome region as in previous studies and the *FAE1* genes were within the range of LD and, therefore, very likely responsible for our identified associations. The small differences to the study of Li et al. (2014) were most likely due to the fact that the SNP in our study were mapped to the recently published *B. napus* genome sequence.

#### 4.2.3. Glucosinolate concentration (GSL)

Plant breeders have strongly reduced the levels of the unhealthy and uneatable glucosinolates in the seeds to be able to use the protein-rich seed cake as an animal feed supplement (Halkier and Gershenzon, 2006). In our study, a number of significantly associated SNPs could be detected which explained even up to 66.1% of the phenotypic variance (Figures 3, 4, Tables 2, 3). Our findings are in accordance with results of previous studies which identified several of the marker-trait associations at the same positions (Basunanda et al., 2007; Feng et al., 2012; Harper et al., 2012; Li et al., 2014; Gajardo et al., 2015).

Hasan et al. (2008) suggested that effective molecular markers for MAS could be used to introduce new genetic variation for low seed glucosinolate content. However, the results of our study suggested that associations which explained high percentages of the phenotypic variation were already present in most of the modern cultivars with alleles which causes low glucosinolate content in the seeds. These low glucosinolate content alleles most likely derived from the strong bottleneck selection for low seed glucosinolate content (so-called double-low, 00, or canola quality) using the low-glucosinolate spring rape cultivar “Bronowski” (Hasan et al., 2008). Nevertheless, the associations GSL.C9.w.2, GSL.w.1, GSL.w.2, GSL.A9.s.1, and GSL.A6.s.1 still have a higher proportion of the undesirable allele in modern cultivars and are promising targets for for MAS.

Several known glucosinolate genes could be mapped near the associations for GSL by BLAST searches (Tables 4, 5). The candidate genes *MAM1* and *MAM3*/*IMS2* (methylthioalkylmalate synthase 1/3) of *A. thaliana* which catalyzes the condensation step of the first three elongation cycles of the Glucosinolate biosynthesis pathway (Kroymann et al., 2001; Textor et al., 2004) were located next to GSL.A2.w.1. Furthermore, the myb transcription factor *ATR1*/*MYB34* of *A. thaliana* controls indolic glucosinolate homeostasis (Celenza et al., 2005) and could be mapped in physical proximity to the associations GSL.A9.s.1 and GSL.A9.w.2. Our findings are in accordance with results of Hasan et al. (2008) who also identified *MAM1* and *ATR1* as potential candidate genes for QTLs of glucosinolate content at these genome positions of *B. napus*. In addition, the myb transcription factor *ATR1* could also be located by a BLAST search next to GSL.C9.w.2 which might be duo to the fact that this is a homolog genome region to the genome region on chromosome A9 (Parkin et al., 2003).

Furthermore, ~200 kb upstream of the *ATR1* transcription factor on chromosome C9 (GSL.C9.w.2) as well as next to GSL.A9.w.4 the HIGH ALIPHATIC GLUCOSINOLATE 1 (*HAG1*) gene (also known as *MYB28*) which is a positive regulator of aliphatic methionine-derived glucosinolates (Gigolashvili et al., 2007b) was localized. This *HAG1* gene was also detected by Harper et al. (2012) and Li et al. (2014) as a candidate gene for glucosinolate content. Next to *ATR1* also *HIG1*/*MYB51* and *HIG2*/*MYB122* are involved in the transcriptional regulation of indole glucosinolate biosynthesis (Gigolashvili et al., 2007a; Frerigmann and Gigolashvili, 2014) and could be mapped to the region of GSL.A9.w.4 at the end of chromosome A9.

Beyond that additional candidate genes like the OBF BINDING PROTEIN2 *OBP2* which upregulates glucosinolate biosynthesis (Skirycz et al., 2006), the cytochrome P450s *CYP79B2* and *CYP79B3* catalyze controlled by the transcription factor *ATR1* (Skirycz et al., 2006) the conversion of tryptophan to indole-3-aldoxime (IAOx) which is a precursor to IAA and indole glucosinolates (Hull et al., 2000; Mikkelsen et al., 2000), and *SUR1* of *A. thaliana* as well as *SUR1-like* of *B. rapa* (Zang et al., 2009) which was characterized as the *C-S* lyase in glucosinolate biosynthesis (Mikkelsen et al., 2004) could be identified. However, all these candidate genes have to be validated in additional approaches like RNA-seq analysis, gene overexpression or gene knockout.

### 4.3. Co-localizing SNP-trait associations

**Figure 5:**
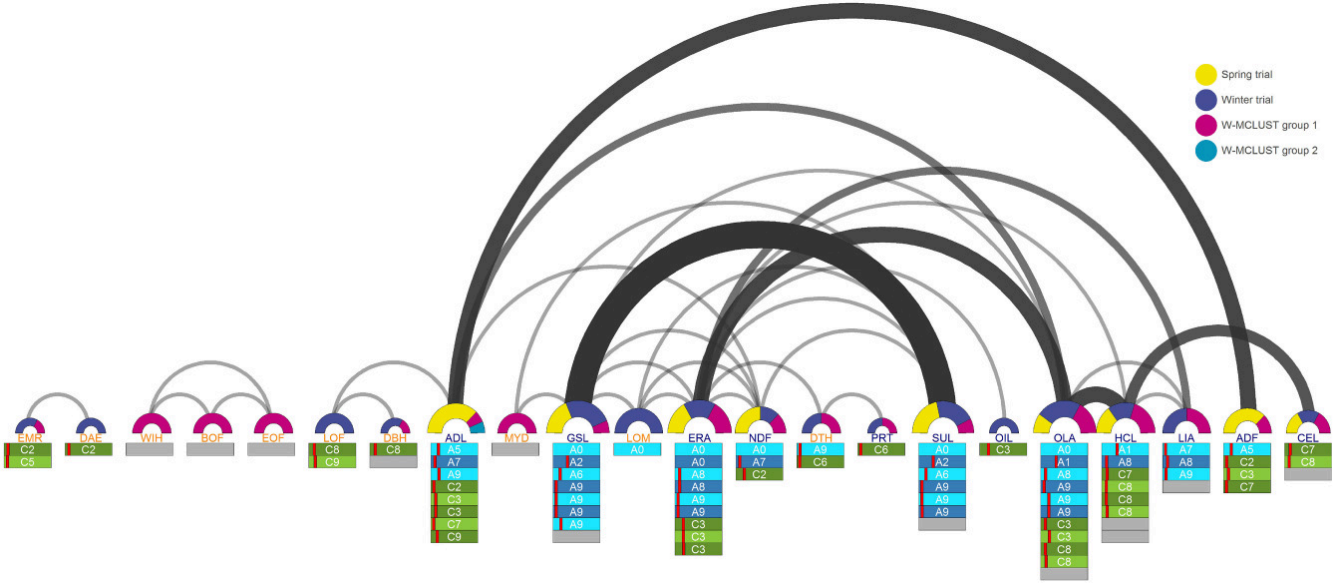
**Arcdiagram of co-localizing SNP-trait associations between two or more traits for the agronomic and seed quality traits**. The width of the arcs as well as the size of the semicircles are related to the number of co-localizing SNP-trait associations between the connected traits. The colors of the semicircles represent the distribution of the co-localizing SNP-trait associations to the spring trial, winter trial, and the WR-MCLUST groups 1 and 2. The bars below the traits symbolize the chromosomes and the red dashes the position of the respective co-localizing SNP-trait associations on these chromosomes. The chromosomes of the A genome are colored blue and the chromosomes of the C genome green. Unknown chromosome positions are colored gray.

**Figure 6:**
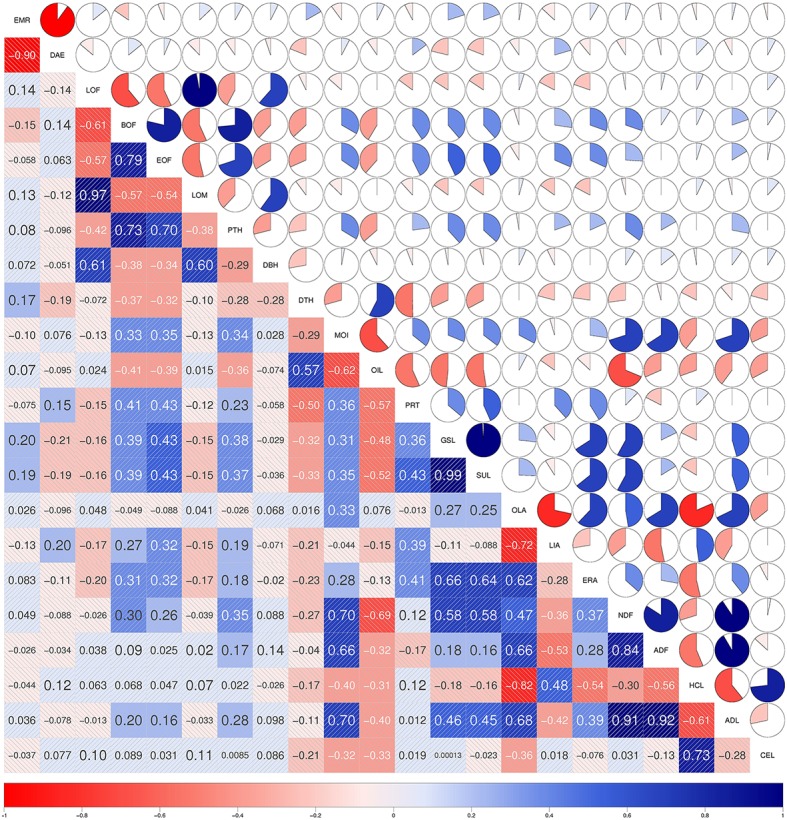
**Correlations of the agronomic and seed quality traits across the 188 inbreds of the spring trial**. In the diagonal panel the traits are listed. In the upper panel the filled portion of the pie and in the lower panel the depth of the shading as well as the font size indicate the magnitude of the correlations. Negative correlations are colored red and positive correlations blue.

**Figure 7:**
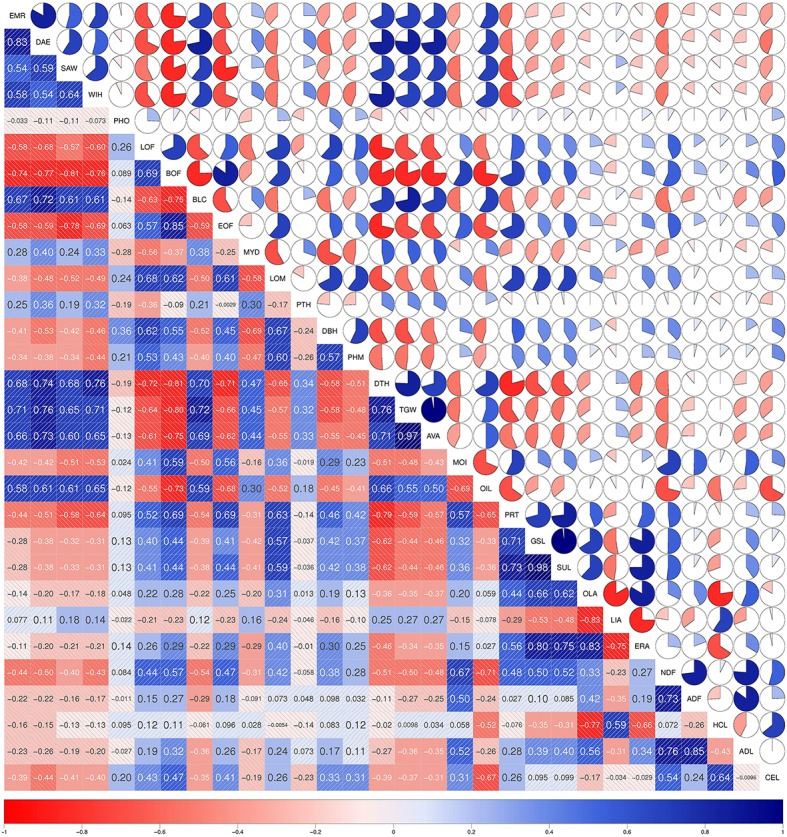
**Correlations of the agronomic and seed quality traits across the 217 inbreds of the winter trial**. In the diagonal panel the traits are listed. In the upper panel the filled portion of the pie and in the lower panel the depth of the shading as well as the font size indicate the magnitude of the correlations. Negative correlations are colored red and positive correlations blue.

We detected 34 SNP-trait associations which co-localized between two or more different traits (Figure 5). For the traits *OLA, ERA, ADL, GSL, HCL*, and *SUL* we found more than six SNP-trait associations which were co-localizing with other traits. The trait pairs with the highest number of identical SNP-trait associations were *GSL*-*SUL, ERA*-*OLA, OLA*-*HCL, HCL*-*CEL*, and *ADL*-*ADF*. With these co-localizing SNP-trait associations, we identified loci which were affecting two or more different traits. These traits, like glucosinolates (*GSL*) which are a group of sulfur-rich secondary metabolites, and the sulfur concentration (*SUL*), were tightly positive correlated between the trait pairs (Figures 6, 7 and Figures S1, S2). Such co-localizing SNP-trait associations can be an advantage in plant breeding if the effect of an allele is beneficial for both traits.

For several co-localizing SNPs associated with the trait pair *GSL*-*SUL* we identified by a BLAST-search the candidate genes *MAM1, MAM3*/*IMS2, SUR1, CYP79B2*, and *CYP79B3* (Tables 4, 5, Tables S3–S6). These glucosinolate biosynthetic genes are all down-regulated by sulfur deficiency and genes controlling sulfur uptake and assimilation are up-regulated (Hirai et al., 2005; Falk et al., 2007). These co-localizing SNP results of the trait pair *GSL*-*SUL* are in good accordance with the fact that glucosinolates may represent up to 30% of the total sulfur content of plant organs (Falk et al., 2007). Thus, the co-localizing *GSL*-*SUL* associations suggested pleiotropic effects or might be due to linkage between the underlying genes, because the extent of LD decays over distances of 677 kb in the *B. napus* diversity set in this study. However, to answer this question conclusively additional approaches like RNA-seq analysis or high resolution fine mapping in segregating populations will be necessary.

## Author contributions

NK, AB, and JL performed the statistical and bioinformatic analyses. IP provided the 6K array data. BW and RS carried out most of the field experiments. NK drafted the manuscript. BS designed and supervised the project. All authors read and approved the final manuscript.

## Funding

This research was funded by the Deutsche Forschungsgemeinschaft and the Max Planck Society.

### Conflict of interest statement

The authors declare that the research was conducted in the absence of any commercial or financial relationships that could be construed as a potential conflict of interest.

## Abbreviations

AEM: adjusted entry mean
BLAST: basic local alignment search tool
DH: doubled haploid
cDNA: complementary deoxyribonucleic acid
GWAS: genome-wide association study
LD: linkage disequilibrium
MAS: marker-assisted selection
MRD: modified Roger's distance
OSR: oilseed rape
PCA: principal component analysis
QTL: quantitative trait loci
RNA: ribonucleic acid
SNP: single nucleotide polymorphism
SSR: simple sequence repeat.
