# Supplementary material for: Agronomic and Seed Quality Traits Dissected by Genome-Wide Association Mapping in Brassica napus
Source: Front Plant Sci. 2016 Mar 31;7:386. doi: 10.3389/fpls.2016.00386 (PMC4814720; doi:10.3389/fpls.2016.00386)
Supplement: Supplementary file 9 [file DataSheet3.pdf]

Supplementary Figures: Agronomic and seed quality traits dissected by genome-wide association mapping in *Brassica napus*

Niklas Körber [1,2], Anja Bus [1,2], Jinquan Li [1], Isobel A.P. Parkin [3], Benjamin Wittkop [4], Rod J. Snowdon [4] and Benjamin Stich [1,*]

[1] *Quantitative Crop Genetics, Max Planck Institute for Plant Breeding Research, Cologne, Germany*
[2] *Institute of Crop Science and Resource Conservation, Plant Breeding and Biotechnology, University of Bonn, Bonn, Germany*
[3] *Agriculture and Agri-Food Canada, Saskatoon, SK, Canada*
[4] *Department of Plant Breeding, Research Centre for BioSystems, Land Use and Nutrition, Justus Liebig University, Giessen, Germany*

Correspondence*:
Benjamin Stich
Quantitative Crop Genetics, Max Planck Institute for Plant Breeding Research, Carl-von-Linné-Weg 10, 50829 Cologne, Germany, stich@mpipz.mpg.de

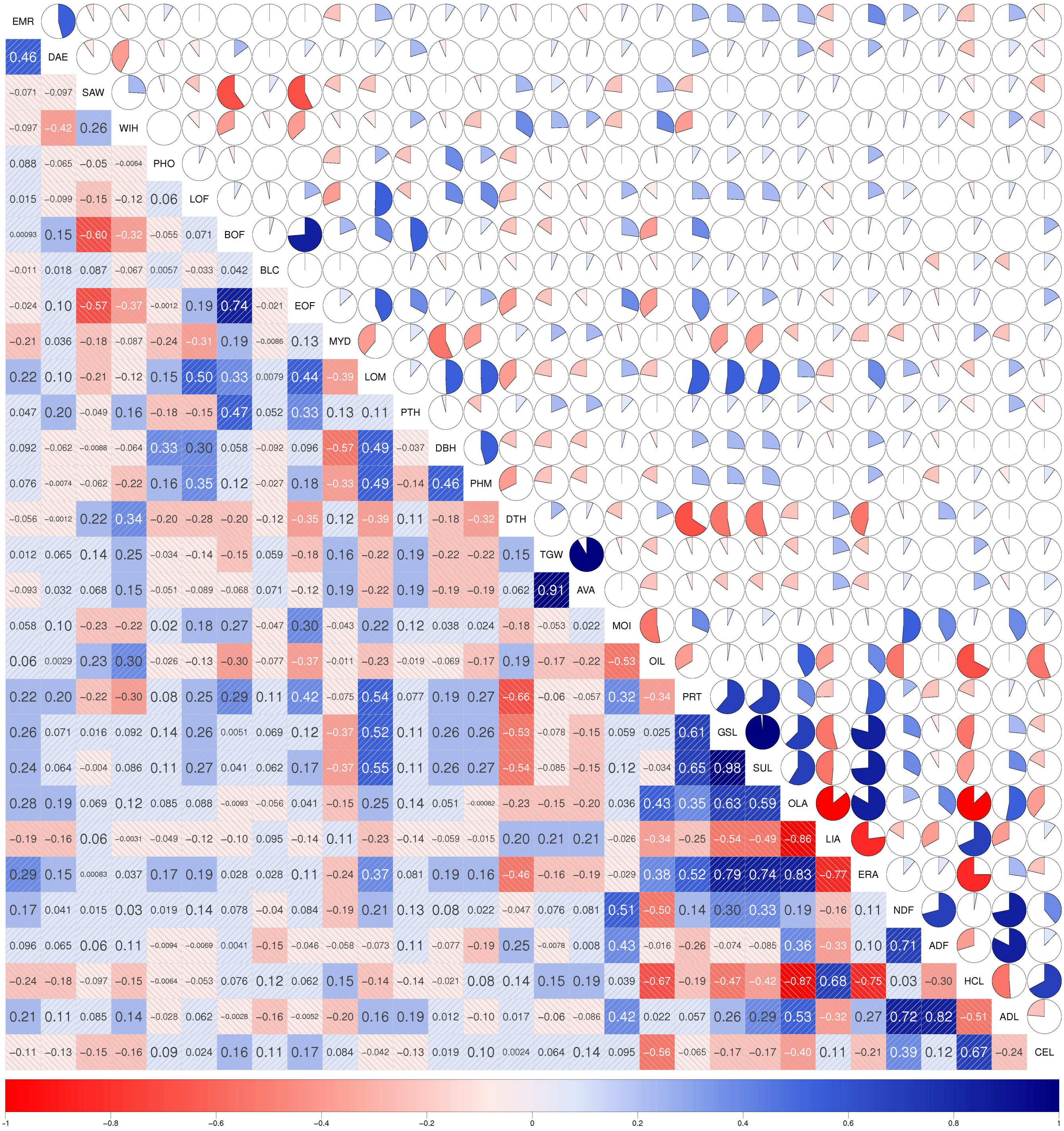

Supplementary Figure 1. Correlations of the agronomic and seed quality traits across the inbreds of the WR-MCLUST groups 1 of the winter trial. In the diagonal panel the traits are listed. In the upper panel the filled portion of the pie and in the lower panel the depth of the shading indicated the magnitude of the correlations. Negative correlations are colored red and positive correlations blue.

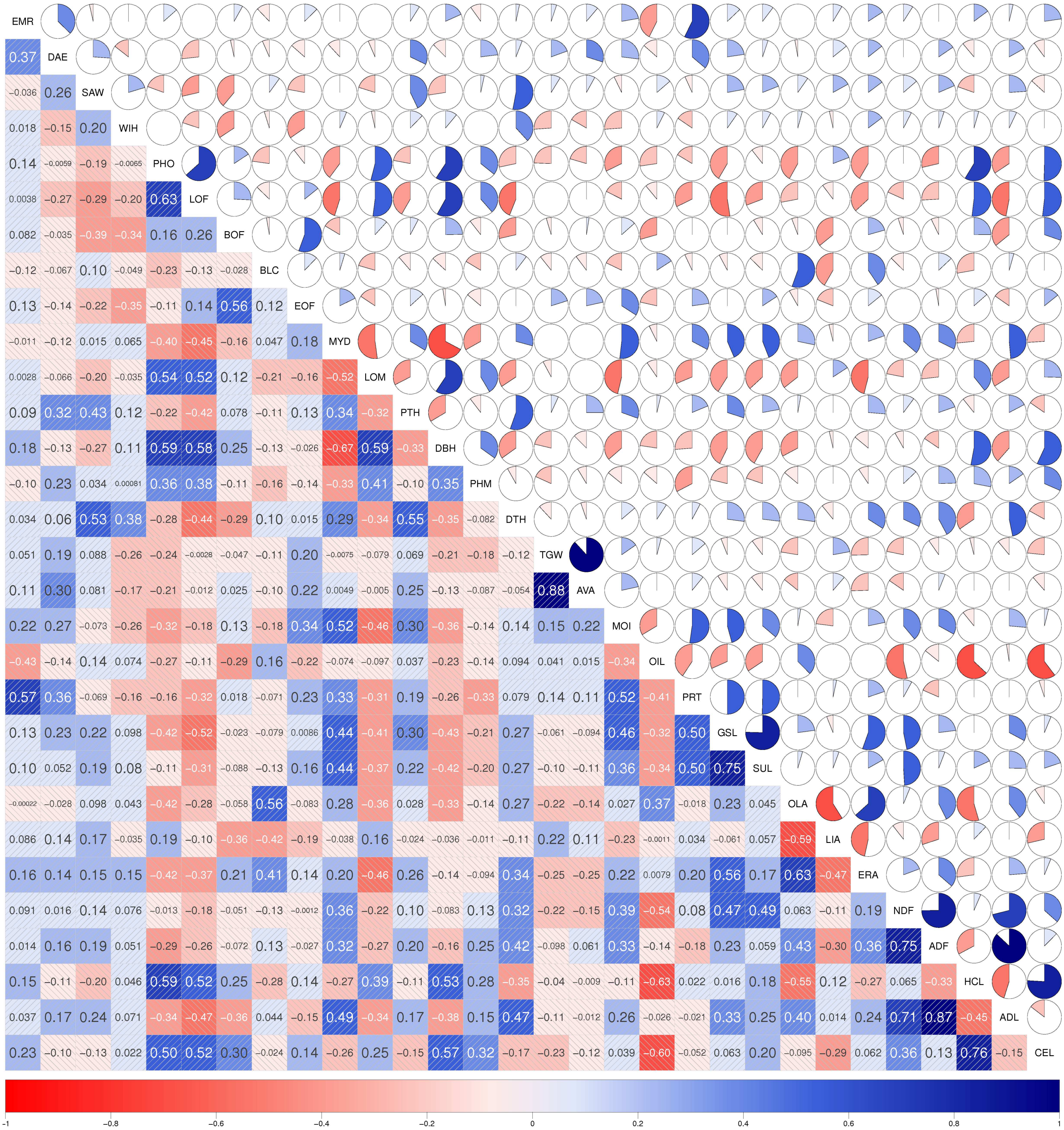

Supplementary Figure 2. Correlations of the agronomic and seed quality traits across the inbreds of the WR-MCLUST groups 2 of the winter trial. In the diagonal panel the traits and candidate genes are listed. In the upper panel the filled portion of the pie and in the lower panel the depth of the shading indicated the magnitude of the correlations. Negative correlations are colored red and positive correlations blue.

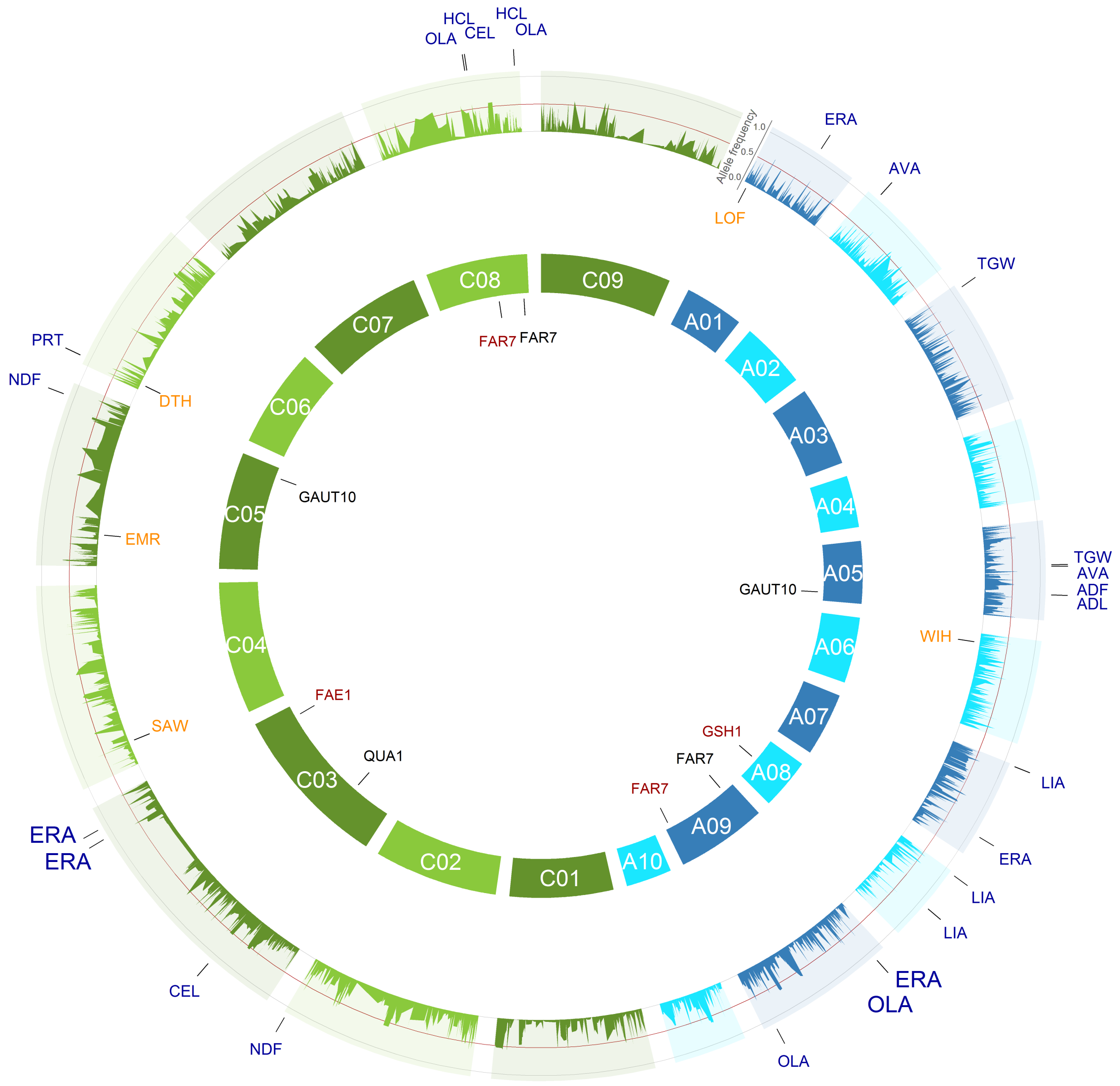

Supplementary Figure 3. All single nucleotide polymorphism (SNP)-trait associations with a *P*-value $< 1.28e\text{-}05$ (Bonferroni correction of α=0.05) identified across the inbreds of the WR-MCLUST groups 1 of the winter trial and their respective positions are marked on the *B. napus* genome. The 3,466 SNPs with their minor allele frequencies in the winter trial are given in the outer circle. The SNPs associated with the agronomic SNP-trait associations are plotted in orange below the allele frequency circle and the seed quality SNP-trait associations in blue outside the allele frequency circle. The size of the letters is related to the proportion of the variance explained by the associations. In the inner circle of the 19 chromosomes, the candidate genes were plotted to their mapping position on the *B. napus* reference genome. The A genome is colored blue and the C genome green.

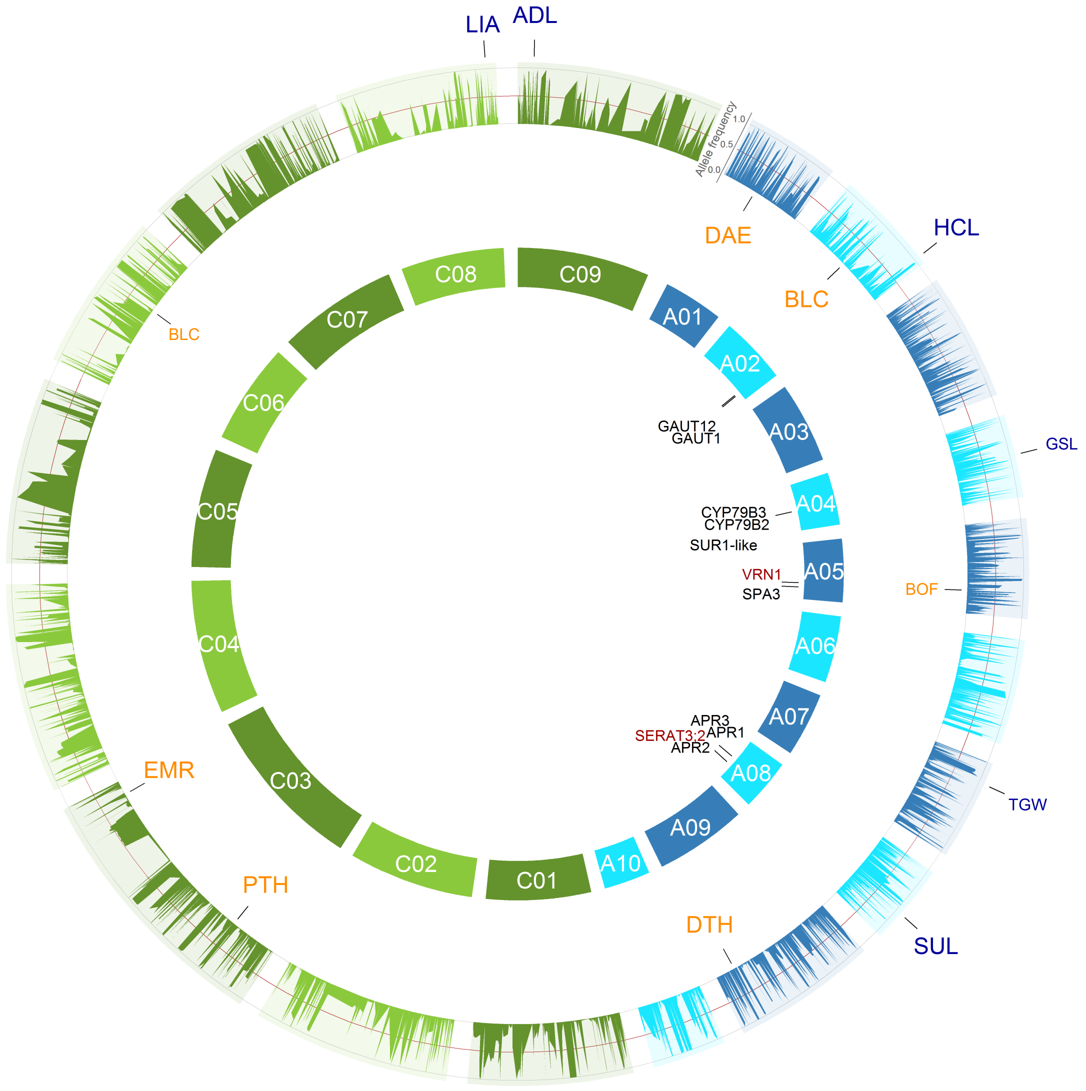

Supplementary Figure 4. All single nucleotide polymorphism (SNP)-trait associations with a *P*-value $< 1.28e\text{-}05$ (Bonferroni correction of α=0.05) identified across the inbreds of the WR-MCLUST groups 2 of the winter trial and their respective positions are marked on the *B. napus* genome. The 3,466 SNPs with their minor allele frequencies in the winter trial are given in the outer circle. The SNPs associated with the agronomic SNP-trait associations are plotted in orange below the allele frequency circle and the seed quality SNP-trait associations in blue outside the allele frequency circle. The size of the letters is related to the proportion of the variance explained by the associations. In the inner circle of the 19 chromosomes, the candidate genes were plotted to their mapping position on the *B. napus* reference genome. The A genome is colored blue and the C genome green.
